# RNA folding and functions of RNA helicases in ribosome biogenesis

**DOI:** 10.1080/15476286.2022.2079890

**Published:** 2022-06-09

**Authors:** Valentin Mitterer, Brigitte Pertschy

**Affiliations:** a Biochemistry Center, Heidelberg University, Im Neuenheimer Feld 328, Heidelberg, Germany; b BioTechMed-Graz, Graz, Austria; c Institute of Molecular Biosciences, University of Graz, Humboldtstrasse 50, Graz, Austria

**Keywords:** Ribosome biogenesis, rRNA folding, snoRNAs, RNA helicases

## Abstract

Eukaryotic ribosome biogenesis involves the synthesis of ribosomal RNA (rRNA) and its stepwise folding into the unique structure present in mature ribosomes. rRNA folding starts already co-transcriptionally in the nucleolus and continues when pre-ribosomal particles further maturate in the nucleolus and upon their transit to the nucleoplasm and cytoplasm. While the approximate order of folding of rRNA subdomains is known, especially from cryo-EM structures of pre-ribosomal particles, the actual mechanisms of rRNA folding are less well understood. Both small nucleolar RNAs (snoRNAs) and proteins have been implicated in rRNA folding. snoRNAs hybridize to precursor rRNAs (pre-rRNAs) and thereby prevent premature folding of the respective rRNA elements. Ribosomal proteins (r-proteins) and ribosome assembly factors might have a similar function by binding to rRNA elements and preventing their premature folding. Besides that, a small group of ribosome assembly factors are thought to play a more active role in rRNA folding. In particular, multiple RNA helicases participate in individual ribosome assembly steps, where they are believed to coordinate RNA folding/unfolding events or the release of proteins from the rRNA. In this review, we summarize the current knowledge on mechanisms of RNA folding and on the specific function of the individual RNA helicases involved. As the yeast *Saccharomyces cerevisiae* is the organism in which ribosome biogenesis and the role of RNA helicases in this process is best studied, we focused our review on insights from this model organism, but also make comparisons to other organisms where applicable.

## Introduction

Ribosomes are the macromolecular machineries that synthesize the cellular proteome by translating the genetic message encoded by mRNAs into polypeptide chains. A small subunit (SSU; 40S in eukaryotes) and a large subunit (LSU; 60S) form the mature (80S) ribosome that consists of 79–80 ribosomal proteins (r-proteins) and four rRNAs. The ribosomal subunits are assembled in an extremely complex biogenesis pathway that takes place in the nucleolus, the nucleoplasm, and finally in the cytoplasm. An efficient and accurate assembly is accomplished by the coordinated activity of at least 200 non-ribosomal assembly factors and around 80 small nucleolar RNAs (snoRNAs), which drive a complicated cascade of rRNA processing, r-protein incorporation, and ribosome maturation steps. Among these assembly factors are, besides several structural proteins, a variety of enzymes such as AAA^+^-ATPases, GTPases, kinases, endo- and exo-nucleases, and RNA helicases. For several assembly factors, the approximate stages of ribosome maturation at which they are required have been determined and their structures when bound to pre-ribosomal intermediates were revealed by cryo-EM. However, the molecular mechanisms how assembly factors facilitate ribosomal restructuring and maturation steps still remain unknown in many cases (for reviews on ribosome biogenesis see [1–6]).

Most of our current knowledge on eukaryotic ribosome assembly comes from studies with the yeast *Saccharomyces cerevisiae*. Despite some differences and additional features owed to increased complexity in higher eukaryotes, there is a high degree of conservation of the assembly pathways from yeast up to humans regarding principal mechanisms and key factors involved [7,8].

In the nucleoli of eukaryotic cells, ribosome synthesis is initiated by the RNA-polymerase-I-driven transcription of a large precursor rRNA (pre-rRNA) species (35S and 47S pre-rRNA in yeast and human, respectively) in which the mature 18S, 5.8S, and 25S (28S in human) rRNAs are separated by internal transcribed spacer elements (ITS) and flanked by 5’ and 3’ external transcribed spacers (ETS) (Figure S1). From this pre-rRNA, mature rRNAs are generated in a highly complex series of RNA processing, modification, and re-arrangement events. The already co-transcriptional association of assembly factors and r-proteins leads to the formation of the first ribosomal precursor particle, the huge (~5 MDa) 90S pre-ribosome (also termed SSU processome) [9–12]. On these first maturation intermediates, the successively associating assembly factors are forming a protective protein scaffold for the evolving unstructured pre-rRNA, which in a stepwise manner is integrated into more mature conformations [13–23]. Already at this co-transcriptional stage, rRNA nucleotide modifications (i.e. 2’-O-methylations and pseudouridylations) are introduced within the nascent 18S rRNA precursor, guided by the association of several snoRNAs (see below) [24,25]. Upon endonucleolytic cleavage at cleavage site A_2_ in ITS1 (Figure S1) [26–28], the first precursors to both the earliest pre-40S and pre-60S particles are born, which from this point on follow independent biogenesis routes.

For the 40S synthesis pathway, the nuclear maturation requires rather few further assembly factor-dependent restructuring steps until pre-40S particles, upon association of export factors, are exported to the cytoplasm [29–32]. In the cytoplasm, decisive re-arrangement and final rRNA processing steps are coupled to quality-control mechanisms ensuring the accurate assembly of the subunit [33–46]. It was suggested that a translation-like cycle, in which the immature pre-40S particles are joined by mature 60S subunits to test-drive the correct assembly of the small subunit, triggers the final endonucleolytic rRNA cleavage step (at site D in ITS1) and the subsequent dissociation of the last remaining assembly factors to complete 40S maturation [47–51].

In contrast to the 40S subunit, a plethora of protein assembly factors joins the nuclear pre-60S particle, which goes through a complex series of maturation events still in the nucleolar compartment [52–56]. Besides that, upon association of snoRNAs with the earliest nucleolar 60S precursors, several rRNA modifications are introduced [25,57]. The coordinated interplay of the transiently-acting assembly factors is subsequently shaping the developing 60S core [53–55] into which also the 5S rRNA species, which is transcribed independently from the other rRNAs by RNA polymerase III, is incorporated [58–62]. Several enzymes such as AAA^+^-ATPases and RNA helicases thereby act as key RNA and protein remodellers on the nascent intermediates, facilitating maturation steps that permit the transition to the nucleoplasmic compartment and later promoting the association of export factors [53,63–74]. After export to the cytoplasm, a further interdependent series of restructuring steps couples assembly factor release with the incorporation of the last missing r-proteins and produces mature 60S subunits [74–84].

## rRNA folding in the course of ribosome biogenesis

**Figure 1.**
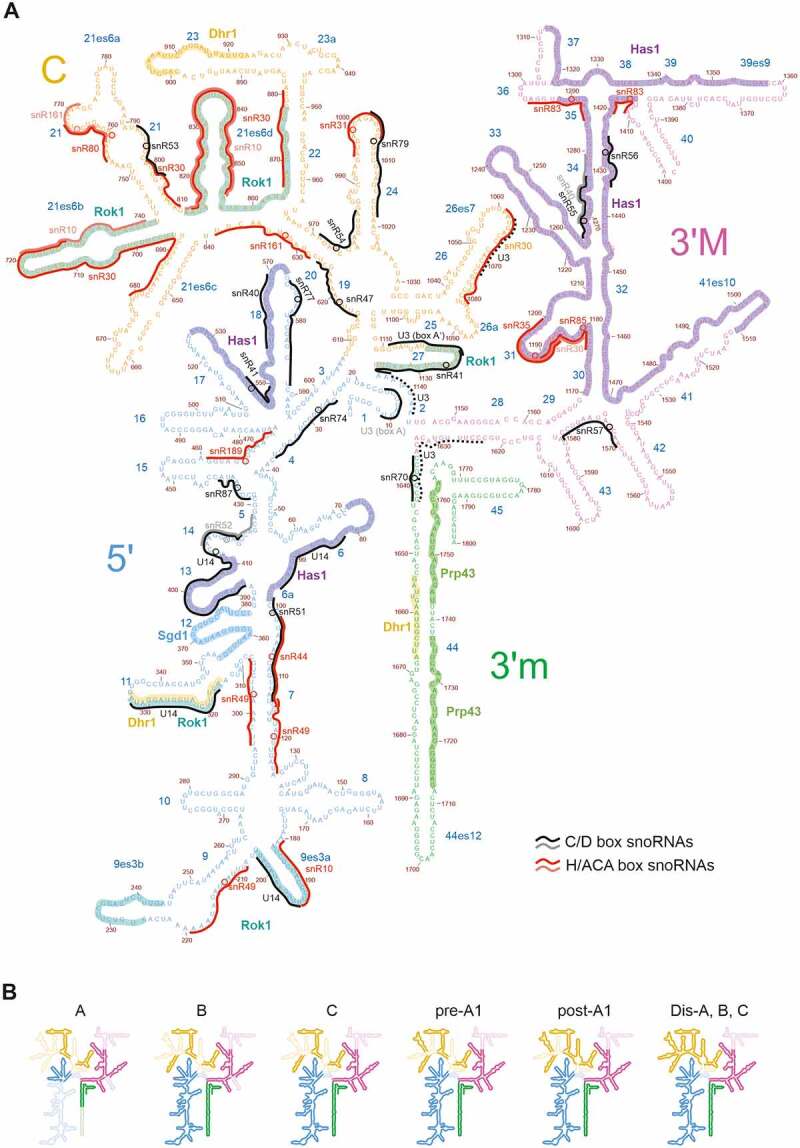
**Secondary structure of the 18S rRNA**. (A) 18S rRNA with the 5’, central (C), 3’ major (3’M) and 3’ minor (3’m) domains indicated in different colours. All known and predicted snoRNA binding sites [13,19,22,23,114,120,121,129,135] are indicated by black/grey (C/D box) or red/pink (H/ACA box) lines, and modification sites are indicated by circles. In the case of the U3 snoRNA, only the hybridization sites observed in cryo-EM structures are indicated in solid lines, while potential additional hybridization sites suggested by biochemical experiments are indicated as dashed lines. The binding regions of RNA helicases, determined by CRAC, and of the Fal1 helicase cofactor Sgd1 [64,135,178,232,238] are indicated. (B) Successive folding of the 18S rRNA [23]. Already folded rRNA elements are displayed in bright colours (unfolded regions in faint colours).

**Figure 2.**
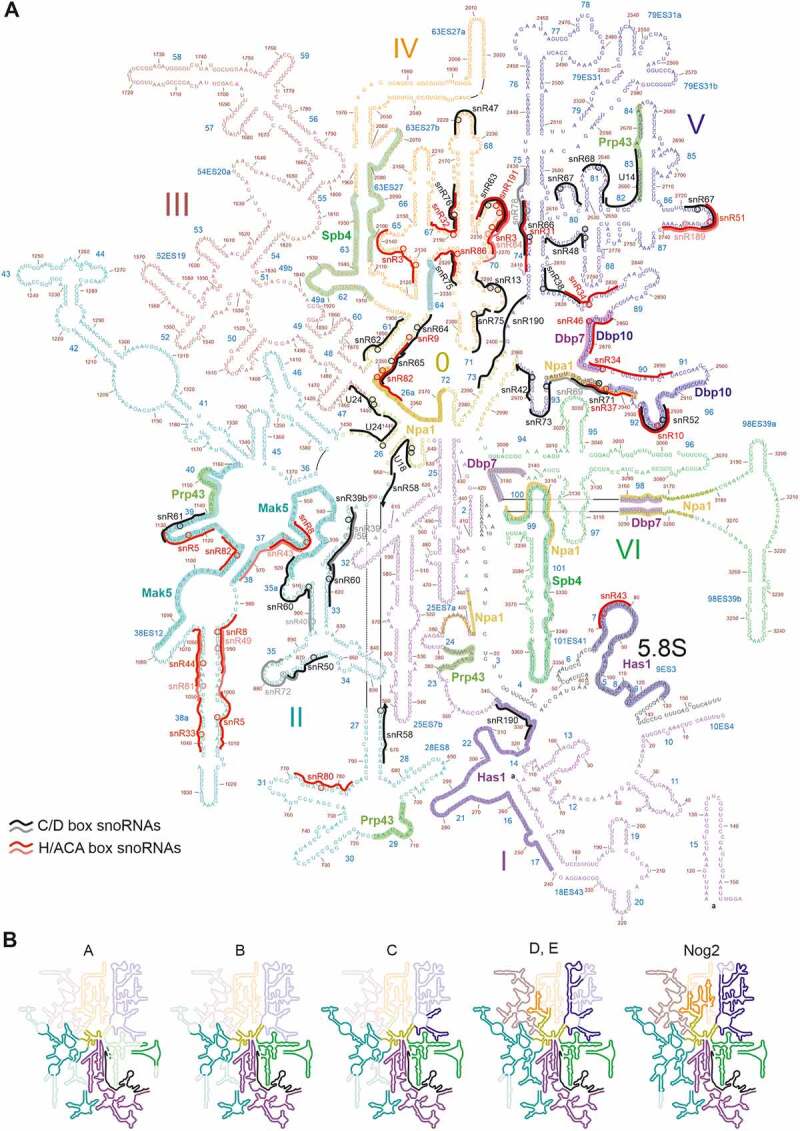
**Secondary structure of the 25S and 5.8S rRNAs**. (A) 25S rRNA with domains 0 to VI indicated in different colours. All known and predicted snoRNA binding sites [114,120,121] are indicated by black/grey (C/D box) or red/pink (H/ACA box) lines, and modification sites are indicated by circles. The binding regions of RNA helicases, determined by CRAC [64,69,232,238,265], are indicated. Additionally, the binding sites of Npa1, an interaction partner of RNA helicase Dbp6 [52], are indicated. (B) Successive folding of the 25S rRNA [54,61]. Already folded rRNA elements are displayed in bright colours (unfolded regions in faint colours).

Both the small and large subunit rRNAs form unique, complex secondary structures composed of several distinct subdomains [85] (see Figure 1 for the yeast 18S rRNA and Figure 2 for the yeast 25S and 5.8S rRNA secondary structures). Bacterial in vitro and in vivo studies as well as in vivo studies in yeast have shed light on the order of domain folding. A breakthrough in recording of the progressive rRNA folding in pre-ribosomal particles came with the advent of high-resolution cryo-EM structures of pre-ribosomal particles. Numerous RNA folds have been identified that change their conformations in the course of pre-ribosomal maturation, as apparent from the structural differences between pre-ribosomes of different maturation stages (see for example [13,18,19,22,23,33,34,40,53–55,59,61,86–91]. In addition to such interpretation of structural differences of elements visible in the cryo-EM structures, also the absence of rRNA segments in cryo-EM structures can be used for conclusions on rRNA folding. This is based on the technical constraints of cryo-EM allowing only to visualize rigid structural elements, while flexible parts fail to be visualized. Hence, the absence of rRNA density corresponding to certain domains can be interpreted as an rRNA domain that has not yet folded into a stable structure. We will only provide a brief overview over the major folding events in ribosome biogenesis, while a more comprehensive summary of the folding events recorded in pre-ribosomal particle structures would make up a review article by itself.

### Folding of the small subunit (SSU) rRNA

**Figure 3.**
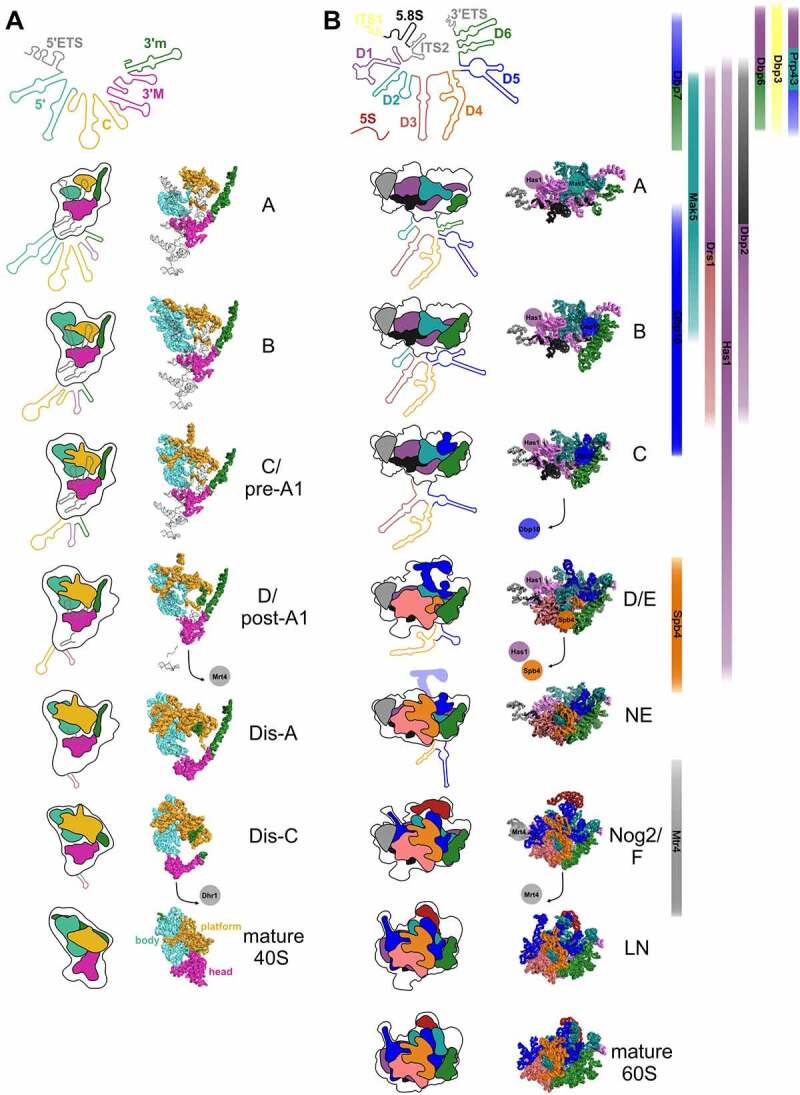
**rRNA folding steps shaping the evolving 90S/pre-40S and pre-60S particles**. 18S (A) or 5.8S, 25S, and 5S (B) (pre-) rRNA domains are colour coded and the consecutive RNA shaping events illustrated schematically (left panels) or using existing cryo-EM structures of ribosomal maturation intermediates (right (A) and middle panels (B)). Association/dissociation of RNA helicases at distinct pre-60S maturation stages is indicated using the colour codes of their potential rRNA target domains ((B), right panel). PDB codes for 90S/pre-40S structures (A) (from top to bottom): 6ZQA (state A), 6ZQB (state B), 6ZQC (state C/pre-A1), 6LQS (state D/post-A1), 6ZQE (state Dis-A), 6ZQG (state Dis-C), 4v88 (mature 40S). PDB codes for pre-60S structures (B) (from top to bottom): 6EM3 (state A), 6EM4 (state B), 6EM1 (state C), 6ELZ (state D/E), 6YLX (state NE – Nop53 early), 3JCT (state Nog2/F), 6YLG (state LN/Rix1-Rea1), 4v88 (mature 60S).

The 18S rRNA folds into four different secondary structure domains: the 5’ domain, the central domain, the 3’ major domain and the 3’ minor domain (Figure 1A). These secondary structure domains also correspond to distinct structural features in the 3D-structure of the SSU, with the 5’ domain forming, together with the 3’ minor domain, the ‘body’, the central domain forming the ‘platform’ and the 3’ major domain forming the ‘head’ domain (Figure 3A).

In vitro reconstitution studies performed with the bacterial SSU suggested that the individual secondary structure domains can fold independently of each other and nucleate from different sites along the rRNA. Importantly, the binding of r-proteins helps the rRNA to fold into the correct conformations. Binding of the so called ‘primary binders’ induces rRNA conformational changes that organize the binding sites for later binding r-proteins, termed ‘secondary and tertiary binders’. Based on the order of r-protein binding, a hierarchical SSU assembly map was established. Usually, proteins bind and re-organize the rRNA in stages. They initially bind weakly; then, interactions are progressively strengthened upon rRNA conformational changes, until the native complex is formed. Hence, rRNA folding promotes r-protein binding and vice versa [92–98].

Although important insights could be gained from these bacterial in vitro studies, it has to be considered that the situation may be different in vivo, as nascent rRNA starts to fold and to recruit assembly factors as soon as it emerges, hence 5’ elements are available earlier than 3’ elements. Of note however, despite the availability of the full-length rRNA, it was observed that also in vitro, an overall 5’ to 3’ order of folding/r-protein assembly is maintained in the bacterial SSU [96,99].

This overall model of SSU assembly could also be transferred to eukaryotes by in vivo investigations in yeast which suggested based on the order of r-protein binding that the SSU body containing the 18S rRNA 5’ domain likely forms before the SSU head containing the 3’ major domain [29]. Such a 5’ to 3’ assembly order was also presumed by two studies mimicking potential intermediates of co-transcriptional pre-ribosome assembly by expressing a series of pre-rRNA fragments with 3’ truncations of different length [15,20].

More detailed information on the order of folding came from yeast, *Chaetomium thermophilum* and human cryo-EM structures of early 90S particles, which showed that 18S rRNA subdomains are kept apart in different regions, confirming their independent maturation [13,14,17,19,22]. The main scaffolds that organize maturation in these early 90S particles are formed by the 5’ ETS and the U3 snoRNA, together with the associated protein complexes UtpA, UtpB, and Mpp10 [13,19,22].

Notably, the structure of the earliest 90S particle that was solved so far challenged the model of a strict 5’ to 3’ order of assembly [21]. In the earliest particles observed in that study (state A), almost 60% of the 3’ major domain as well as of the 3’ minor domain was visible, while this was the case only for ~35% of the central and ~30% of the 5’ domain (Figures 1B and 3A). Based on these observations, the authors suggested a reverse order of folding of the 18S rRNA domains, with the 3’ domain and the central domain starting to fold before the 5’ domain [21]. Nevertheless, in lack of even earlier structures, it is still unclear in which order the domains visible in that structure get folded. More work on the earliest pre-ribosomal particles, ideally by combining cryo-EM with chemical structural RNA probing methods (which would also provide information about the structural status of rRNA elements not visible in the cryo-EM structures), would be helpful to better dissect the earliest SSU rRNA folding steps.

Apparently, although the earliest particles have a higher proportion of the 3’ domains folded compared to the 5’ and central domains, the following maturation steps focus on further folding of the 5’ parts of the 18S rRNA. In subsequent 90S intermediates observed, already the entire 5’ domain was folded, while folding of the 3’ domains had not yet further progressed compared to the earliest particles described above (Figures 1B and 3A, state B). Along that line, ~2/3 of the r-proteins binding to the body were visible in these structures, but only few r-proteins binding to the head domain (~1/5) [13,14,19,21,22]. To conclude, in the initial maturation steps, the 3’ domains are faster in adopting stable folding of a higher proportion of their RNA helices, but the 5’ domain is the first in which folding of the rRNA is completed.

In the above described early 90S particles, the 5’ ETS is still bound to 90S particles, even after separation from the 18S rRNA by cleavage at the processing site A_0_. Subsequently, the 5’ ETS, which folds into ten helices (H1 to H10) and two additional helices base-pairing with U3 snoRNA (Ha and Hb), is successively degraded by the nuclear exosome, with only helices H1 and H2 and the elements base-pairing with U3 being visible in the post-A_1_ structure. Dismantling of the 5’ ETS goes along with the stepwise removal of 90S assembly factor modules, generating binding sites for newly binding proteins, as well as exposing box A of the U3 snoRNA and the endonuclease Utp24, which catalyzes cleavage at site A_1_ [18,23,86,100,101]. In the course of transition from pre-A_1_ to post-A_1_ particles, also most parts of the U3 snoRNA become detached. Nevertheless, U3 is still present in the earliest pre-40S particles, with box A binding to helices H1 and H27 of the 18S rRNA [23,86]. In the process of transition from the pre-A_1_ cleavage state to a 90S intermediate in which the 5’ ETS has already been cleaved at site A_1_, additional helices in the central domain, particularly in expansion segment 6 (helix 21), become accommodated, as well as helix 42 (H42) in the 3’ major domain (Figures 1B and 3A, states B1 to post-A_1_,) [23,86].

In the subsequent maturation stages Dis-A, B, C, almost all 18S rRNA sequence elements are already folded, with the exception of two regions: 18S rRNA helices H35 to H40 in the 3’ major domain; and helices H2 and H27 who’s base pairing is prevented as the U3 snoRNA is still present (Figures 1B and 3A) [23,86].

Subsequently, U3 is released by the Dhr1 helicase (see below), allowing for the central pseudoknot (CPK), a long-range tertiary interaction representing a key structural feature of the 18S rRNA, to form [23,102]. Last but not least, helices H35 to H40 in the 3’ major domain, which are kept in an immature state by assembly factor Rrp12, successively fold and re-arrange in the course of further maturation of pre-40S particles [33,87,90,91].

### Folding of the large subunit (LSU) rRNA

The 25S rRNA folds into six distinct secondary structure domains (named in the 5’ to 3’ direction as domains I to VI), and a seventh domain (domain 0) from which these domains originate (Figure 2A) [85]. Moreover, the 5.8S rRNA base-pairs with 25S rRNA domain I (Figure 2A). In contrast to the SSU, where the secondary structure domains correspond to distinct structural features in the 3D-structure, the secondary structure elements of the LSU are more intertwined (Figure 3B).

Bacterial LSU assembly was not characterized to the same extent as SSU assembly in vitro. Still, a hierarchical binding map of r-proteins was also established for the LSU [103,104]. Moreover, a bacterial cryo-EM study of 60S subunits stalled in maturation suggested a modular assembly, with distinct blocks of rRNA secondary structures maturing separately from the other blocks [105].

Yeast studies provided, comparable to SSU assembly, also evidence for a hierarchical assembly of r-proteins to the LSU [106,107]. Structural probing studies demonstrated that the earliest LSU specific pre-rRNA in yeast, the 27SA_2_ pre-rRNA, is highly flexible and that this flexibility is greatly reduced upon transition to the 27SB pre-rRNA [107,108]. Moreover, domains I and II, as well as the 3’ 25S rRNA domain VI were already in close to mature conformations in 27SA_2_ pre-rRNA containing pre-60S particles, whereas domains III, IV, and V adopted a similar to mature conformation only in the later 27SB containing pre-60S particles [108]. In line with the high flexibility observed in chemical probing, no structures of 27SA_2_ containing pre-60S particles have been solved until now. The earliest pre-60S particles, for which a cryo-EM structure could be solved, contain 27SB pre-rRNA and display stably folded cores of 25S rRNA domains I (with the 5.8S rRNA) and II, as well as ITS2 (together with its associated factors forming the prominent pre-60S foot structure), in state 1/A particles (Figures 2B and 3B) [54,55]. The next domain adopting a stable folding state is domain VI in state 2/B particles, which further enlarges the solvent-exposed back side of the subunit [54–56]. Thereafter, further helices of domain II and initial parts of domain V are compacted (state C particles), followed by larger parts of domain V and parts of domains III and IV, which results in the formation of the polypeptide exit tunnel (PET) at this stage (states D and E) [54]. During the transition to the nucleoplasm (states NE1 and NE2), a re-arrangement of the characteristic L1 stalk (domain V helices H75-H78) into a near-mature conformation goes along with further folding in domains IV and V, which leads to stabilization of the majority of the LSU rRNA domains in Nog2-particles (Figures 2B and 3B, state F) [54,61]. Additionally, also the 5S rRNA, which is initially incorporated at an early 60S assembly stage, becomes stably integrated and structurally visible as part of the now compacted central protuberance (CP) (domain V helices H80-H87) on these nucleoplasmic pre-60S particles [59,61]. At these intermediates, also further elements on the intersubunit side of the LSU, including first parts of the immature peptidyl-transferase centre (PTC) become evident [61]. Subsequently, pre-60S particles undergo a massive remodelling by removal of the ITS2-containing foot and rotation of the 5S RNP into its final orientation completing CP construction in Rix1-Rea1 particles (Figure 3B, state LN) [53,61,74]. Further folding steps, particularly at the intersubunit side and the PTC of the pre-60S particle occur very late in maturation, after their export into the cytoplasm [74,78,82,83].

Notably, despite this order of LSU domain folding, the intertwined nature of the domains suggests that folding of the individual subdomains is more cooperative than SSU folding. Indeed, many LSU r-proteins, but also ribosome assembly factors, bind to two or more different secondary structure domains, hence their binding may help to establish tertiary contacts between different domains.

### Mechanisms to prevent misfolding and promote correct folding

Due to co-transcriptional folding, the 5’ end of the rRNA can already start folding before more 3’ sequences have even been synthesized. This entails that there is some delay from the time an rRNA segment is synthesized until the time the complementary segment it is destined to base-pair with becomes available. Many secondary structure features are formed by short-range interactions and the time delay until the sequence for base-pairing is synthesized is short. However, there are also secondary structure elements that form by base-pairing of distant regions. Especially in case of the first helices of each domain, termed root helices, the rRNA elements base-pairing with each other can be up to 600 nucleotides apart (Figures 1A and 2A).

Importantly, it has been shown that while local helices arising from short-range interactions can form alone in vitro in Mg^2+^ containing buffer, formation of secondary structures requiring long-range interactions depends on proteins [109]. The reason for that is probably that in the absence of the cognate base-pairing sequence, rRNAs can undergo mispairing with other, yet unpaired rRNA regions. Consequently, initial secondary structures may need to be re-organized to allow for the formation of long-range interactions [99]. Studies in bacteria suggest that RNA-binding proteins can prevent such misfolding [99,110–112]. Functions of proteins in rRNA folding likely include the protection of single-stranded sequences from mispairing, the improvement of the kinetics of re-folding of misfolded elements or the distortion of RNA structures to allow for the formation of the correct long-range interactions.

Recent studies provided in depth insights into the role of r-proteins in co-transcriptional rRNA folding in bacteria [99,111,113]. R-proteins uS4 and uS7 bind to helices of the 5’ domain and the 3’ major domain of 16S rRNA, respectively, which are both formed by base-pairing of distant rRNA regions. While uS4 and uS7 were able to bind to short transcripts designed to contain only the base-paired 5’ and 3’ sequences of their binding site (without the sequence in between), they were not able to stably bind to their natural rRNA binding sites co-transcriptionally. The most likely explanation for these observations is that in the co-transcriptional scenario, the 5’ rRNA element undergoes mispairings and that even after synthesis of the 3’ rRNA element, it takes some time until the mispairings are resolved and the correct base pairs are formed [111,113]. This further supports the model that rRNA initially misfolds. Importantly however, stable co-transcriptional association of uS4 and uS7 with their binding sites was achieved when in addition, nearby r-proteins were added, suggesting cooperativity of r-protein recruitment and rRNA folding events [111,113].

Although the exact role of individual proteins on rRNA folding was not investigated in such molecular detail in eukaryotes, it is conceivable that most of the proteins associating with rRNA at early maturation stages, be it r-proteins or assembly factors, have an impact on rRNA folding and can therefore be regarded as RNA chaperones. Importantly, archaea and eukaryotes employ not only proteins, but also trans-acting RNAs in rRNA folding, namely small nucleolar RNAs (snoRNAs).

## snoRNAs and their function in rRNA folding

snoRNAs are short non-coding RNAs that assemble with a distinct set of proteins into snoRNPs. Most snoRNPs introduce modifications into the rRNA. There are two main classes of snoRNPs: H/ACA snoRNPs, which catalyse pseudouridylation, and C/D box snoRNPs, which promote 2’-O ribose methylation [25]. In both types of snoRNPs, the snoRNA base-pairs with the target site in the rRNA and thereby selects the modification site. In H/ACA snoRNPs, the pseudouridine synthase Cbf5 then converts the target uridine into pseudouridine, whereas in C/D box snoRNPs, the methyltransferase Nop1 methylates the target nucleotide. In yeast, 29 different H/ACA snoRNPs mediate pseudouridylation of 14 different uridines in the 18S rRNA and 30 uridines in the 25S rRNA, while 46 different C/D box snoRNPs introduce 17 methylations into 18S rRNA and 37 methylations into 25S rRNA (Figures 1A and 2A) [114] (snoRNA database: https://people.biochem.umass.edu/fournierlab/3modmap/main.php).

Importantly, methylations and pseudouridylations can alter the properties of RNA by either favouring or blocking the formation of base-pairs, and can consequently have local effects on RNA folding [115,116]. Moreover, 2’-O ribose methylation can shift the equilibrium between the C3’-endo and C2’-endo sugar pucker conformations of ribose towards the C3’-endo conformation, which rigidifies the RNA backbone [117,118].

Besides the effects of the introduced modifications on the rRNA structure, a probably more important role of snoRNAs in rRNA folding comes from their base-pairing with rRNA elements which are double-stranded in the mature ribosome. Hence, the hybridization of a snoRNA to a given region most likely prevents the folding of the respective region.

It is remarkable that almost all so far known/predicted snoRNP binding sites on the 25S rRNA (Figure 2A) map to only four subdomains, namely domains 0, II, IV and V. As discussed above, domain 0 is the central hub connecting all other domains via root helices, and the sequences that base pair to form these root helices are very distant in the primary structure, hence it is obvious that at least the 5’ portions of these sequences need to be protected from mispairing until the 3’ portion is synthesized.

Also, the binding of numerous snoRNAs to domains IV and V, which are the two last 25S rRNA domains to be finally folded, might be indicative of a role of snoRNAs as RNA chaperones coordinating these folding events by preventing premature base pairing events. An example for a critical region in the rRNA for which this may be relevant is the PTC, comprised of 25S rRNA helices H89 to H92 in domain V, which is targeted by nine different snoRNAs (Figure 2A [119]. It is tempting to speculate that besides the reported function of these snoRNAs in introducing modifications that fine-tune translation [119], they additionally also contribute to the correct and timely folding of the PTC structure.

25S rRNA domain II is as well bound by many snoRNAs (Figure 2A) and some of them bind in late-folding parts of this domain, like helices H33, H35 and H38a. Interestingly, however, most snoRNAs bind to parts of domain II, which are already folded in the earliest particles for which cryo-EM structures exist, like helices H27, H31, H32, H37, H38, H39 and H40. Hence, snoRNAs binding in these regions are the ones that have to perform their function and dissociate again in the earliest steps of pre-60S maturation, in order to allow these parts of domain II to fold in time.

It is more complicated to draw correlations between the order of folding and the binding of snoRNAs in SSU maturation. snoRNA binding sites are more distributed in the 18S rRNA (Figure 1A) and, moreover, the earliest folding steps in SSU maturation are not as well resolved on the structural level as in LSU maturation.

Experimental evidence based on the ‘CLASH’ (crosslinking, ligation and sequencing of hybrids) method suggests that some snoRNAs have, beside their hybridization to the site where they guide modifications, additional binding sites in the rRNA [120,121]. An example is snR40, that functions as a methylation guide in 18S rRNA helix H34, but was also found to base-pair with helix H18 [121]. Such additional base-pairings likely serve structural functions. Examples for snoRNAs with known or presumed functions as RNA chaperones are:

### U3 snoRNA

The U3 snoRNA is a large C/D box snoRNA. It is one of the few essential snoRNAs and does not catalyse any rRNA modification but is instead required for early pre-rRNA processing steps at sites A_0_, A_1_ and A_2_ [122]. The U3 snoRNP is a core element of 90S particles and is the only snoRNA that could so far be visualized bound to pre-ribosomal particles [13,19,21–23]. Apart from the common C/D box proteins Nop1, Nop56, Nop58 and Snu13, the U3 snoRNP contains another protein component, Rrp9 [13,19,22,123] and is in contact with multiple additional proteins, including the Imp3/Imp4/Mpp10 complex, via interaction with Imp3 [13,19,22]. Indeed it was shown that Imp3 and Imp4 are important for U3 binding, by mediating formation of duplexes with pre-rRNA [124]. Cryo-EM structures also allowed to visualize the base-pairings of U3 snoRNA with the 18S rRNA (Figure 1A) and the 5’ ETS [13,19,22]. These structural data suggest that the 5’ and 3’ hinges of the U3 snoRNA hybridize to the 5’ ETS RNA, while the box A’ and box A regions hybridize to 18S rRNA helices H1 and H27, both in the region of the CPK. Also earlier biochemical analyses had suggested that U3 base-pairs with two regions in the 5’ ETS and two regions in the 18S rRNA that later form the CPK [125–130]. Notably, while both biochemical and structural data agree on the binding of U3 snoRNA to H1, the hybridization to H27 observed in the structures was not observed in biochemical analyses, which proposed U3 hybridization to the adjacent H2 [129]. Moreover, based on CLASH analyses, additional binding sites in proximity, one in H26 and one in the H28/H44 hinge, were predicted [121]. These additional sites were not observed in cryo-EM structures, however their positioning in proximity to the elements later forming the CPK makes it likely, that these represent true hybridization sites of U3 that are formed during distinct pre-ribosomal maturation steps.

Regardless of whether or not additional rRNA elements apart from H1 and H27 are bound by U3, the binding of the snoRNA in this important region is thought to function in holding these subdomains of the 18S rRNA apart, preventing their premature interaction and hence regulating the timing of CPK formation (see Dhr1 section below).

A second major function of U3 snoRNA is connected to its base-pairing with the 5’ ETS: the coordination of early folding and cleavage events at sites A_0_, A_1_ and A_2_ in the pre-rRNA [128,129,131,132]. Cryo-EM data indicate that U3 and associated proteins keep the pre-rRNA cleavage site A_1_ amenable to cleavage by the Utp24 endonuclease [13,18,19,22].

### snR30

snR30 is a H/ACA snoRNA and represents another essential snoRNA that is required for early rRNA cleavages at sites A_0_, A_1_ and A_2_ [133]. snR30 binds to several sites in expansion segment 6 (ES6), corresponding to H21 of the 18S rRNA (Figure 1A) [134,135]. As ES6 is not visible and hence likely unfolded in early 90S structures (Figure 1B) [13,19,22], snR30 may function in preventing premature ES6 folding, which in turn was hypothesized to influence rRNA processing and/or the recruitment of assembly factors [133].

### snR10

snR10 is non-essential, but its deletion leads to a cold-sensitive phenotype [136]. It has two functions, which are mediated by separate domains of the snoRNA [137]: it directs pseudouridylation at U2923 in 25S rRNA and it additionally functions in 18S rRNA synthesis, where its deletion results in rRNA processing defects at sites A_0_, A_1_ and A_2_ [138]. snR10 was found to bind to the 5’ ETS [137]. Moreover, binding sites of snR10 in 18S rRNA H21 (ES6) were identified, which overlap with snR30 binding sites, suggesting that these snoRNAs hybridize to the 18S rRNA at different time-points (Figure 1A) [135].

### U14/snR128

U14 is, beside U3 and snR30, the third essential snoRNA. It is a C/D box snoRNA required for early pre-rRNA cleavages at sites A_0_, A_1_ and A_2_. Like snR10, U14 potentially has two different functions: it has a non-essential region that mediates methylation in 18S rRNA H14, whereas the second region is essential and binds to H6 of the 18S rRNA [135,139–141]. Additionally, binding sites of U14 in 18S rRNA helices H9 and H11 were identified (Figure 1A) [135].

### snR190

Although snR190 is predicted to guide methylation at G2395 in 25S rRNA, this modification has never been detected; therefore, snR190 was suggested to function in rRNA folding instead [52]. In addition to its 25S rRNA binding site in the domain V root helix H73, snR190 was predicted to also base-pair with H4 in domain I (Figure 1A) [52]. Based on that, it was suggested that snR190 could function as chaperone for formation of the root helix of domain V and to function in drawing together domains I and V [52]. snR190 is additionally required for the stable association of Npa1 complex members Rsa3 and Nop8 with pre-60S particles [142].

### snoRNA release

Given that snoRNAs often prevent rRNA folding events, rRNAs have to be liberated from the snoRNAs again at some point to allow them to fold into their destined conformation. It is only poorly understood how snoRNAs are released, however, several mechanisms can be envisaged: snoRNAs may be displaced by similar mechanisms as upon remodelling of mispaired rRNA segments; binding of assembly factors or r-proteins may compete snoRNAs away from their binding site; last but not least, several RNA helicases have been implicated in actively mediating snoRNA release [25].

## RNA helicases: overview

RNA helicases are enzymes that utilize NTPs (mostly ATP) to bind and remodel their RNA substrates. Their activities are essential for almost every cellular process involving RNA and include, among others, the disassembly of RNA duplexes, RNA strand annealing, re-arrangement of RNA-protein complexes, and unwinding of sn(o)RNAs [143,144]. RNA helicases usually share the P-loop Walker A motif I and downstream Walker B motif II, essential for NTP binding and hydrolysis, respectively [145,146]. Based on additional characteristic sequence motifs and common structural and biochemical properties they are classified together with DNA helicases and divided into six superfamilies (superfamilies SF1 to SF6) [147,148]. SF2 contains the largest number of RNA helicases including the helicases relevant for ribosome biogenesis addressed in this review (i.e. the DEAD-box, DEAH-box, Ski2-like families) [149]. RNA helicases of SF2 are characterized by a common, structurally almost identical, catalytic core consisting of two globular highly similar RecA-like domains (resembling the fold of the bacterial RecA recombination protein) that are connected by a short flexible linker [147,149–153]. This helicase core contains at least twelve distinct signature motifs required for ATP-binding and hydrolysis, substrate RNA-binding and remodelling activity (**Figure S2**). While most motifs required for ATP-binding and hydrolysis (including Walker A motif I and Walker B motif II) are found in the N-terminal RecA-like domain (RecA1), the C-terminal RecA-like domain (RecA2) mainly harbours motifs for substrate RNA-binding. Regarding the structural conformation of the helicase core two major states are distinguished, while also several transition states are suggested to exist [144,152,154–156]. In the absence of ligands, the two RecA-like domains are in an inactive open conformation, showing relatively high flexibility to each other. Initial binding of ATP to RecA1 together with (substrate) RNA binding induces the active closed helicase conformation in which the two core domains are tightly connected and form an ATPase active site accommodating the ATP in a cleft between the two RecA-like domains. ATP-hydrolysis and product release is ultimately re-inducing a (temporal) domain opening resulting in release of the remodelled substrate or inducing another cycle of ATP-binding and substrate processing, dependent on the type of RNA helicase.

An overview of all RNA helicases in ribosome biogenesis, including their (putative) functions, is provided in Table 1. The majority of RNA helicases involved in ribosome biogenesis belong to the DEAD-box family (e.g. Rok1, Has1, Dbp10), which is named after the common Asp-Glu-Ala-Asp amino acid signature sequence in motif II [157]. With 37 members in humans and 26 members in yeast, DEAD-box helicases also represent the largest RNA helicase group within SF2 [158]. Besides the catalytic helicase core, they typically contain flanking N- and/or C-terminal auxiliary domains that, in contrast to some other helicase families, are not well conserved among different DEAD-box helicases. The auxiliary domains exhibit a variety of potential functions, including modulating helicase activity, and provide specificity to helicases by interacting with specific RNA and protein binding partners [159–161]). In general, the ATP-dependent RNA unwinding by DEAD-box helicases occurs by a local strand displacement mechanism. Thereby the helicase core is directly loaded onto its RNA duplex substrate and duplex melting occurs in a non-processive way without translocation of the helicase or RNA [100,162–168]. This strand displacement occurs in single ATP-dependent cycles of ligand binding and release events and is limited to shorter RNA duplex regions. In this non-processive RNA unwinding model, closure of the two RecA-like domains upon ATP- and RNA-duplex binding forces bending of the substrate RNA that, due to steric constraints, results in local base-pair melting and finally strand separation. Subsequent ATP-hydrolysis brings the helicase core back to the open conformation going along with the release of the unwound RNA strands.

**Table 1.** RNA helicases in ribosome biogenesis and their presumed functions

Name	Type	Maturation step	Cofactors	Known or hypothesized function in RNA folding/unwinding
Fal1	DEAD	90S	Sgd1	unknown
Dbp8	DEAD	90S	Esf2	unknown
Dbp4/Hca4	DEAD	90S	unknown	18S rRNA 5’ domain folding; U14 release
Rrp3	DEAD	90S	unknown	unknown
Rok1	DEAD	90S	Rrp5	snR30 release from 90S particles; Rrp5 release from 90S particles
Dhr2	DEAH	90S, (early pre-40S)	unknown	unknown
Dhr1/Ecm16	DEAH	90S, early pre-40S	Utp14	promotion of A_1_ cleavage; release of the U3 snoRNA from the 18S rRNA, which triggers formation of the central pseudoknot
Prp43	DEAH	90S, pre-40S, pre-60S	Pfa1, Pxr1 (G-patch)	snoRNA (snR72, snR60, others) release; 18S rRNA H44 restructuring
Has1	DEAD	90S, early to late nucleolar pre-60S	unknown	U14 release; Rrp36 release; contact formation between 25S rRNA domain I and 5.8S rRNA
Mtr4	Ski2-like	90S, nucleoplasmic pre-60S	unknown	5’ETS and ITS2 unwinding and recruitment of the nuclear exosome for 3’-5’ degradation of these spacer elements
Dbp6	DEAD	(90S), early nucleolar pre-60S	unknown	unknown
Dbp7	DExD	(90S), early nucleolar pre-60S	unknown	snR190 (possibly also snR34) release
Dbp9	DEAD	early nucleolar pre-60S	unknown	unknown
Dbp3	DEAD	early nucleolar pre-60S	unknown	snoRNA release; ITS1 re-arrangement
Drs1	DEAD	early/intermediate nucleolar pre-60S	unknown	promotion of A_2_ cleavage; stable Nop7**–**Erb1**–**Ytm1 incorporation
Mak5	DEAD	early/intermediate nucleolar pre-60S	unknown	25S rRNA H39 restructuring
Dbp2	DEAD	early/intermediate nucleolar pre-60S	unknown	U8 snoRNA release in higher eukaryotes; re-arrangement of ITS2 hairpin in yeast; both are proposed to regulate formation of the proximal 25S(28S)/5.8S stem
Dbp10	DEAD	intermediate nucleolar pre-60S	unknown	25S rRNA H89-H92 (PTC) folding
Spb4	DEAD	late nucleolar pre-60S	unknown	25S rRNA H62/H63 restructuring

A second important RNA helicase group for ribosome biogenesis is the DEAH-box family, including assembly factors Prp43, Dhr1, and Dhr2. The helicase core of this group, which is named after the Asp-Glu-Ala-His signature in motif II, contains in large parts a functionally similar set of conserved motifs as found in DEAD-box helicases, however, with a clear difference in their primary amino acid sequences [147,149]. Another distinction is that DEAH-box helicases typically share a conserved C-terminal extension of the catalytic core consisting of a winged-helix (WH), a ratchet-like helical bundle (HB), and an oligosaccharide-binding (OB) fold domain (**Figure S2**) [152,155]. By tight interactions with the RNA-binding surface of the helicase core, this C-terminal extension regulates substrate binding and plays a crucial role in the catalytic cycle for coupling NTP hydrolysis with RNA unwinding activity [155,169–172]. At the mechanistic level, DEAH-box RNA helicases are translocating enzymes, which are usually processing and thereby disrupting base-pairs of their RNA substrates in a 3’ to 5’ direction [155,170,172–176]. Their helicase core does not attach to structured RNA duplexes but instead depends on single-stranded overhangs for its initial loading. RNA unwinding typically requires multiple NTP binding and hydrolysis steps going along with opening and closure of the DEAH-box helicase core. The resulting translocation of the substrate by pulling it through the core channel in principle also allows the disassembly of RNA structures buried deeply within RNPs such as pre-ribosomal intermediates.

## RNA helicases in the SSU processome/90S particle maturation

### Fal1

Fal1 is a nucleolar DEAD-box helicase with homology to translation initiation factor eIF4A that is required for 40S synthesis. Its depletion, as well as overexpression of a dominant negative mutant blocks the early A_0_, A_1_ and A_2_ processing steps [177–180]. Fal1 binds, in a relative transient manner, to 90S particles [178,181,182] and directly interacts with assembly factor Sgd1, which contains a MIF4G domain, also found in eI4F4G, an interaction partner of the translation initiation RNA helicase eIF4A [178]. The MIF4G domain of Sgd1 is required for interaction with Fal1 and stimulates its ATPase activity in vitro [178]. While the binding site of Fal1 on 90S particles could not be identified due to the transient nature of its interaction, the Sgd1 rRNA binding site could be mapped by CRAC to 18S rRNA H12 (Figure 1A). Moreover, crosslinking-mass spectrometry suggested an interaction of Sgd1 with Lcp5, a protein binding in proximity to H12 [13]. Considering the direct interaction of Fal1 with Sgd1, Fal1 may also bind in the region of H12 and Lcp5. Still, the function of Fal1 at this site remains elusive.

### Dbp8

Dbp8 is another DEAD-box helicase that localizes to the nucleolus [183]. Dbp8 directly interacts with the ribosome assembly factor Esf2, and this interaction stimulates its ATPase activity in vitro [184]. Dbp8 and Esf2 associate with 90S particles [10,19,177,184]. Dbp8 depletion, as well as over-expression of a dominant negative Dbp8 mutant blocks early pre-rRNA processing steps at sites A_0_, A_1_ and A_2_ [184,185]. The exact function of Dbp8 in 90S particles is however up to now unknown.

### Dbp4 may promote re-arrangements around U14 and U3 binding regions at the 18S rRNA 5’ domain

Dbp4 (also known as Hca4) was found associated with 90S pre-ribosomes purified via Pwp2-TAP [186]; however, as it is not a stoichiometric component of such precursor particles the interaction might be rather transient or unstable [10]. The DEAD-box helicase is essential for cell growth and its cellular depletion generates a 40S synthesis defect with accumulation of the 35S and 20S pre-rRNAs and decreased production of the 27SA_2_ pre-rRNA [186,187]. The helicase hydrolyses ATP and unwinds short 10 nucleotide RNA duplexes in vitro [188,189]. While in both *dbp4* motif I (Walker A) and motif III (SAT) mutants the ATPase activity was strongly reduced, these mutants are not dominant upon over-expression [186]. Thus, ATP binding and/or hydrolysis by Dbp4 could potentially be a prerequisite for its pre-ribosomal binding.

Depletion of Dbp4 results in strongly increased co-sedimentation of the U14 and, to a lesser extent, of the snR41 snoRNAs with 90S fractions after sucrose gradient centrifugation [186]. Since U14 was accumulating also on affinity-purified 90S particles upon Dbp4 depletion, whereas snR41 levels were decreased, it was speculated that U14 could be a direct helicase target and its activity would allow snR41 recruitment in a following maturation step [186]. In line with a possible role of Dbp4 in U14 dissociation, the helicase was found to act as multi-copy suppressor for 18S rRNA synthesis defects yielded by mutations in the yeast-specific essential Y motif of U14 [190]. While trapping of U14 within 90S fractions was reproduced in another study, the authors did not observe any direct co-precipitation of the snoRNA in Dbp4 immunoprecipitations but instead some amounts of U3 [187]. Furthermore, trapping of U14 in 90S fractions occurred as well upon U3 and assembly factor Mpp10 depletion [187], and also upon Has1 and Dbp8 depletion [191]. Vice versa, U14 depletion resulted in increased 90S association of U3 and further snoRNAs [187]. DDX10, the human Dbp4 homolog, was also shown to act on 90S pre-ribosomes and was associated with a U3 snoRNP complex that did not contain U14 [192].

A yeast two-hybrid assay revealed interaction of Dbp4 with assembly factor Bfr2 and the helicase co-sedimented in a complex (without U3 and U14) with Bfr2 and Enp2, which are part of the Kre33 module on compacted 90S pre-ribosomes after integration of the 18S rRNA 5’ domain [21,193]. Potentially, Dbp4 could associate simultaneously with Bfr2**–**Enp2 to the 18S rRNA 5’ domain to which also U14 hybridizes [19,140]. Consistently, Dbp4 was shown to get recruited to the nascent 5’ domain at a similar maturation stage as the Enp2**–**Bfr2**–**Lcp5 module and the Dbp8 co-factor Esf2 [20].

Considering all data, it remains doubtful that Dbp4 would directly facilitate U14 snoRNA release. However, Dbp4 may promote folding around the U14 binding region in the 18S rRNA 5’ domain, resulting in integration of this domain into the 90S scaffold, which was shown to occur only after initial integration of the 3’ and central domains [21]. Additionally, the helicase re-arrangement activity may affect the folding of the U3 binding site of the 18S rRNA 5’ domain as well.

### Rrp3

Rrp3 is a DEAD-box protein that shows in vitro ATPase activity in the presence of single-stranded RNA and was shown to unwind short RNA duplexes with single-stranded extensions in vitro in an ATP-binding, but not hydrolysis-dependent manner [188,189]. Its depletion results in a 40S synthesis defect due to defects in early pre-rRNA processing steps, particularly at sites A_1_ and A_2_ [179,194]. Rrp3 is a component of 90S particles [177,179,182], but the function of Rrp3 in these particles has not been characterized yet.

### Rok1 is required for release of Rrp5 and snR30

Rok1 is a DEAD-box helicase and shows ATPase activity in vitro [188,195]. Rok1 can unwind short RNA duplexes with single-stranded extensions (while it fails to unwind longer duplexes), a function that is dependent on ATP binding but not hydrolysis [189]. Another study suggested that Rok1 preferentially binds double stranded RNA, stabilizes duplex formation, and promotes RNA annealing in vitro. This annealing activity is conducted by the ADP bound state of the protein [196].

Rok1 is a nucleolar protein and is required for early pre-rRNA processing steps at A_0_, A_1_ and A_2_ [197]. Rok1 binds to several elements of the 18S rRNA including H21 (ES6B and D), H9, H11, but also H27 in proximity of the CPK (Figure 1A) [135].

The *ROK1* gene is a high copy suppressor of *rrp5* mutants [198], suggesting that the functions of Rok1 and the 90S and pre-60S assembly factor Rrp5 are connected. Notably, while the C-terminal domain of Rrp5 functions in SSU synthesis, its N-terminal domain is involved in LSU synthesis [199–201]. Both Rrp5 domains bind to RNA elements in ITS1. The Rrp5 C-terminal domain moreover shares a binding site with Rok1 in H21 ES6D, and additionally, both Rrp5 and Rok1 bind to regions in proximity to the CPK [135,201].

Rrp5 directly interacts with Rok1 via its C-terminal domain [196] and is required for recruitment of Rok1 to 90S particles [202]. Moreover, Rrp5C enhances RNA duplex annealing by Rok1 [196]. Last but not least, the activity of Rok1 is required for the release of Rrp5 from 90S particles [203]. As Rrp5 has an additional function in early pre-60S particles, it was proposed that release of Rrp5 from the 18S rRNA allows Rrp5 to remain bound to the LSU specific part of ITS1 after A_2_ cleavage and to subsequently carry out its function in pre-60S maturation [203].

Besides that, Rok1 also has a snoRNA related function: Rok1 crosslinks to snR30 [135], as does Rrp5 [201]. Additionally, both Rok1 and Rrp5 crosslink to H21 ES6, the region to which snR30 hybridizes (Figure 1A), demonstrating the strong link of Rok1 and Rrp5 to the snR30 snoRNA [135,201]. Last but not least, Rok1 depletion, but also inhibition of its ATPase activity leads to a massive accumulation of snR30 on pre-ribosomes [191]. All these results suggest a function of Rok1 in the release of snR30.

snR30 also accumulates in ribosome bound-form upon expression of a Rrp5-mutated variant that binds less efficiently to Rok1 and is consequently less efficiently released from 90S particles. Based on that result, and the observation that Rok1 was still recruited to pre-ribosomal particles in that mutant, the authors claimed that Rok1 only has an indirect role in snR30 release by promoting Rrp5 release, which they in turn postulated to be a prerequisite for snR30 release [203]. However, the phenotypes of the *rrp5* mutant could also be explained retaining the model that Rok1 releases snR30: Rrp5 is a cofactor of Rok1, and was reported to provide specificity to Rok1 by changing its conformation [196]. Therefore, Rok1 is likely not fully functional when Rrp5 is mutated, which would explain the less efficient release of snR30 by Rok1 in that mutant. Future studies will have to address whether snR30 is a direct release target of Rok1 or not.

Apart from snR30, Rok1 also crosslinks to the U14, U3 and snR10 snoRNAs [135]. Notably, all these snoRNAs were found to hybridize to 18S rRNA regions in proximity to Rok1 binding sites (Figure 1A). CLASH analyses also revealed a potential U14 snoRNA binding site that overlaps with the Rok1 crosslinking site in H11 (Figure 1A) [135], and also Rrp5 has a binding site in this area [201]. Moreover, CLASH data suggest that beside their main binding sites in other regions, both snR10 and U3 snoRNA also base-pair with H21 ES6 (Figure 1A) [135]. There is also genetic evidence for a close connection between Rok1 and snR10, as *rok1* mutants were found to be synthetic lethal with mutations of the H/ACA snoRNA snR10 and of the gene encoding H/ACA snoRNP component Gar1 [197]. Despite all these connections of Rok1 to U14, U3 and snR10, Rok1 is not required for their release, as Rok1 depletion does not lead to the accumulation of these snoRNAs in pre-ribosome bound form [191]. Hence, the interaction of Rok1 with these snoRNAs may serve a structural role instead.

### Dhr2

Dhr2 is a DEAH-box helicase that is localized in the nucleolus. Its depletion, as well as overexpression of a dominant-negative mutant inhibits pre-rRNA processing at sites A_0_, A_1_ and A_2_ [179,204]. Moreover, Dhr2 was shown to associate with the SSU processome [179] and to interact with the SSU processome factor Nop19 [205,206]. Sucrose gradient sedimentation analyses showed that Dhr2 normally sediments in the 90S range and in the soluble fractions. In contrast, upon Nop19 depletion, no soluble Dhr2 fraction is visible anymore but instead, Dhr2 sediments in the range of 40S subunits [205]. These data suggest that Nop19 may be required for Dhr2 release. Moreover, Dhr2 may be present during the 90S to pre-40S transition and may upon Nop19 depletion be trapped before it is released from early pre-40S particles. Dhr2 directly interacts with the nucleolar deubiquitylating enzyme Ubp10, but whether this interaction is relevant for ribosome biogenesis is up to now unclear [207,208].

### Dhr1 removes the U3 snoRNP, thereby promoting central pseudoknot formation

Dhr1 is a DEAH helicase and shows RNA-dependent ATPase and RNA unwinding activity in vitro [102]. ATP binding but not ATP hydrolysis is required for RNA unwinding by Dhr1, suggesting that ATP binding is important for duplex unwinding, while ATP hydrolysis causes product release [102]. Dhr1, as well as its human ortholog DHX37 have a co-factor, Utp14, which directly binds to Dhr1/DHX37 and activates its RNA unwinding activity [209,210]. While human UTP14 was reported to also stimulate ATPase activity of DHX37 [209,211], yeast Utp14 does not activate Dhr1 ATPase activity in vitro [210].

Besides its requirement for pre-rRNA processing in 90S particles, Dhr1 is responsible for release of the U3 snoRNA from pre-ribosomal particles, and thereby promotes rRNA re-arrangements leading to the formation of a key structural element of the SSU, the CPK. The mechanisms of Dhr1 recruitment and function are summarized below.

#### Dhr1 recruitment/re-positioning

Biochemical as well as structural data indicate that Dhr1/DHX37 initially binds to 90S particles and remains bound during the transition to the earliest pre-40S particles [18,23,102]. Split-tag affinity purification via Dhr1 and Noc4 yielded (in addition to early pre-40S subunits) 90S particles in different maturation stages representing particles before and after processing at cleavage site A_1_, suggesting that Dhr1 is recruited to 90S particles at an early stage, before A_1_ cleavage. Nevertheless, Dhr1 failed to be visualized in cryo-EM structures of pre-A_1_ 90S particles, suggesting it is bound to flexible elements in these particles [23]. Dhr1 is composed of an N-terminal extension, followed by a DEAH helicase module, and a C-terminal domain including an extension, which is not found in other DEAH-box RNA helicases [212]. While the N-terminal extension was shown to interact with Bud23 [213,214] the helicase core interacts with Utp14 [210]. Utp14 and Bud23 together were proposed to be required for the efficient recruitment of Dhr1 to 90S particles [102,210]. As however, Dhr1 is not visible in structures of the earliest particles to which it is bound to, and only small parts of Utp14 are visible, while Bud23 has not yet been observed in 90S structures at all, cryo-EM structures do not provide any further insight into the roles of Bud23 and Utp14 in the initial recruitment of Dhr1. Recently, it was suggested that Bud23 might instead bind to the SSU precursors even later than Dhr1, and assist Dhr1’s function within early pre-40S particles [215].

**Figure 4.**
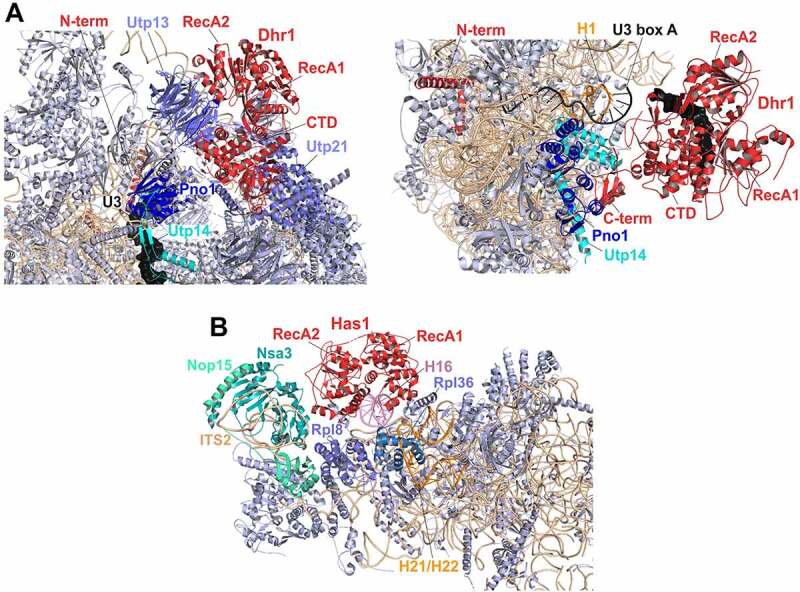
**Cryo-EM structures of helicases Dhr1 and Has1 bound to their pre-ribosomal substrate particles**. (A) Dhr1 (red) bound to 90S (left panel, PDB: 6ZQD) or pre-40S (right panel, PDB: 6ZQG) particles. The assembly factors Utp14 (cyan) and Pno1 (blue) in proximity to the Dhr1 C-terminus, as well as the U3 snoRNA (black) and 25S rRNA H1 (Orange, right panel) base-pairing with U3 box A are depicted. (B) Has1 (red) binds to pre-60S intermediates (PDB: 5Z3G) on top of the ITS2-containing foot structure close to Nsa3. The two RecA-like domains interact with H16 (purple) of 25S rRNA domain I. The Has1 25S rRNA crosslink site at H21/H22 (Orange) and further proximal assembly factors and -r-proteins are indicated.

The first 90S structures in which Dhr1/DHX37 could be visualized are post-A_1_ 90S particles [18,23] (Figure 4A, left panel). CRAC data already suggested that Dhr1 binds to 18S rRNA helices H11, H23, and H44 (Figure 1A) [102]. Indeed, contacts of the Dhr1/DHX37 N-terminal extension with these (as well as additional) RNA elements could also be observed in the yeast (H11) and human (H11, H23 and H44) post-A_1_ 90S structures [18,23].

In contrast to the extensive rRNA contacts formed by the N-terminal extension, the Dhr1/DHX37 helicase and C-terminal domains are not in contact with RNA in post-A_1_ 90S particles. Instead, the C-terminal domain of Dhr1 interacts with assembly factors of the UTP-B complex. Notably, this position of the C-terminal domain is in earlier (pre-A_1_) 90S particles occupied by Pno1. Hence, the Dhr1 helicase core and C-terminal domain have to be at another position in pre-A_1_ particles and likely move to the site previously occupied by Pno1 in the course of transition from pre- to post-A_1_ 90S particles. Considering the multiple RNA contacts of the Dhr1/DHX37 N-terminal extension, it is tempting to speculate that the N-terminal part of the protein represents an initial anchoring point, whereas the helicase core and C-terminal domain move in the course of 90S maturation.

In post-A_1_ particles, the catalytic and C-terminal domains of Dhr1 are distant from the U3 substrate, while part of the N-terminal domain of Dhr1/DHX37 is positioned in proximity to the helicase target site in the region where 18S rRNA H27 base-pairs with box A’ of the U3 snoRNA (Figure 4A, left panel) [18,23].

Importantly, during the transition to pre-40S particles, the N-terminal domain remains unchanged, while the helicase and C-terminal domains of Dhr1 are repositioned a second time and are recruited by Utp14 and Pno1 into closer proximity of the unwinding substrate, box A of the U3 snoRNA, which is base-paired with 18S rRNA H1 (Figures 1A and 4A, right panel) [23]. This recruitment via Utp14 is only possible because before that, the segment of Utp14 responsible for Dhr1 recruitment is relocated to a site in proximity to the U3 snoRNA [18,23].

#### The function of Dhr1 in 90S particles

In structures of yeast and human 90S particles, Dhr1/DHX37 is in an inactive, open conformation [18,23]. Several mechanisms have been suggested to ensure that Dhr1/DHX37 remains inactive in these early particles: (1) A similar open conformation was observed in a recent high-resolution crystal structure of the (ADP-bound) Dhr1 helicase core, in which an autoinhibitory loop within the RecA2 domain that blocked the substrate channel was observed [18]. Although the resolution of 90S cryo-EM structures was not sufficient to visualize this autoinhibitory loop, the same relative domain orientations in these structures suggest that the open conformation may be maintained by this auto-inhibitory loop of Dhr1/DHX37 also in 90S particles [18]. (2) An N-terminal extension of Pno1 was observed that reaches to the Dhr1 catalytic domain in post-A_1_ 90S particles and was proposed to block binding of Dhr1 to the RNA substrate [23]. (3) In 90S particles, Dhr1/DHX37 is still physically distant from its U3 substrate [18,23]. (4) Utp14 is not yet in the correct position to recruit nor to activate the helicase in these particles [18,23].

The catalytically inactive conformation of Dhr1 in 90S particles suggests that Dhr1’s role in 90S particles is a structural one that does not rely on its catalytic function. Dhr1 depletion inhibits pre-rRNA cleavages at sites A_1_ and A_2_ [19,204] and prevents the transition from 90S to pre-40S particles, suggesting that Dhr1 has a function in 90S maturation and/or the 90S to pre-40S transition [19,86]. Enp1-TAP particles purified after Dhr1 depletion contain greatly increased amounts of 33S and 22S pre-rRNAs, both representing intermediates in which A_1_ cleavage has not occurred [19]. Hence, the physical presence of Dhr1 is important for A_1_ cleavage.

This goes in line with structural data: in the course of exosome-mediated 3’ to 5’ removal of the 5’ ETS after A_0_ cleavage, a part of the 5’ ETS clamped between Utp7 and Pwp2 has to be set free. This happens by a change of the orientation of the two proteins, leading to a ‘ring-opening’. Additionally, a repositioning of Pno1 and the associated 18S rRNA H45 to a site previously occupied by Krr1 and Faf1 takes place (while the position liberated by Pno1 is subsequently occupied by the Dhr1 catalytic domain). The re-positioned Pno1/H45, additionally bound by Dim1, is then stabilized by the Dhr1 N-terminus [216]. These repositioning events also have the consequence that the U3 box A, the later substrate of Dhr1, which was previously constrained, is liberated. In contrast, in the absence of Dhr1, the above-described maturation steps do not take place: Instead, Dhr1 depletion traps 90S particles in a stage in which a 5’ ETS segment is still positioned between Utp7 and Pwp2, and additionally, Pno1/H45 have not completed the movement to their new position and are trapped next to Pwp2 [86,216]. Hence, Dhr1 is required for important repositioning events which are necessary so that maturation proceeds to A_1_ processing.

In contrast to the consequences of complete absence of Dhr1, an ATP-binding incompetent *dhr1* mutant is able to perform the 90S to pre-40S transition (with the mutated Dhr1 remaining bound to pre-40S particles), and accumulates 21S pre-rRNA, an intermediate in which A_1_ cleavage has occurred, while A_2_ cleavage was skipped [102]. Hence, ATP binding by Dhr1 seems to be required to allow for normal A_2_ cleavage to occur. This observation is further complemented by data showing that 90S, but also pre-40S particles purified by split-tag purification via both Noc4 and Dhr1 contain a substantial fraction of 21S pre-rRNA [23]. A_2_ cleavage normally occurs within 90S particles, but potentially, when Dhr1 does not exert its function in A_2_ cleavage in time (e.g. in the ATP-binding deficient mutant as well as in a small sub-population of wild-type particles), some particles may escape this step, resulting in pre-40S particles with the pre-rRNA not yet cleaved at site A_2_.

#### Dhr1 function in early pre-40S particles

Dhr1 as well as its human counterpart DHX37 directly bind to the U3 snoRNA, as revealed by co-IP, CRAC analyses and cryo-EM [23,102,204,211]. Strong biochemical and genetic data demonstrate that Dhr1 functions in U3 snoRNA release: U3 snoRNA accumulates bound to pre-40S particles in *dhr1* catalytic mutants and *dhr1* cold-sensitive mutants. Moreover, the growth defects of the cold-sensitive mutants can be rescued by mutations in the U3 region base-pairing with 18S rRNA, suggesting that weakening of the interaction of U3 with 18S rRNA can partially compensate for the absence of active Dhr1 [213]. Furthermore, the pre-40S-trapped U3 snoRNA in the *dhr1* ATP-binding deficient mutant still remains base-paired with the 18S rRNA [102]. As detailed above, this base-pairing of U3 snoRNA with the 18S rRNA prevents CPK formation, hence in turn, the release of U3 snoRNA directly regulates the timing of the formation of the CPK. In line with that, r-protein Rps2, which binds to the CPK, is absent from the pre-40S particles accumulating in this *dhr1* mutant, further underscoring that the CPK does not form in the absence of the unwinding activity of Dhr1 [102].

As described above, Utp14 is re-positioned in the course of 90S to pre-40S maturation. In early pre-40S particles, the Dhr1 C-terminal domain docks to the repositioned region of Utp14 and is thereby targeted into closer proximity of the U3-18S rRNA hybrid (Figure 4A, right panel). In these particles, Dhr1 was observed in a substrate-bound conformation [23]. The conformation resembled the conformation in a crystal structure of the human Dhr1 ortholog DHX37 in a substrate bound, but nucleotide free state, that was suggested to correspond to the conformation of a nucleotide exchange intermediate prior to binding to the next ATP molecule [23,209]. The switch from the open state observed in 90S particles to this more closed state was proposed to be the consequence of different states of the ATP binding/hydrolysis cycle [23]. Additionally (or alternatively), the autoinhibitory loop observed in the open state may determine the switch between the open and closed conformation of the helicase [18].

Despite the deep insights gained from these recent structural studies, the exact mechanism of dismantling of U3 snoRNA from the pre-rRNA was still not fully unravelled. One reason is that the part of Utp14 required for activation of the helicase [209] is not visible in the Dhr1-containing pre-40S particles [23]. Moreover, even though Dhr1 was found in an RNA bound conformation in pre-40S particles, the U3-rRNA hybrid to be disrupted is still distant from the substrate binding region of Dhr1 in this structure [23]. It was proposed that through successive rounds of ATP binding and hydrolysis, Dhr1 would successively draw the RNA closer and finally dismantle the U3 snoRNA from the 18S rRNA [23].

## RNA helicases in 90S and 60S particle maturation

### Prp43 is directed to different functions via G-patch proteins

Prp43 is a DEAH helicase with multiple functions. It participates in mRNA splicing where it mediates intron lariat release from the spliceosome, and additionally functions in different steps of ribosome biogenesis. The splicing related function of Prp43 is not discussed further here, as this goes beyond the scope of this article.

Prp43 harbours RNA-dependent ATPase and helicase activity [217,218]. It unwinds RNA duplexes with a 3’ overhang much more efficiently than duplexes with a 5’ overhang, indicating that Prp43 displays 3’ to 5’ directionality [219]. The function of Prp43 is regulated by a group of co-factors termed G-patch proteins (reviewed in more detail in [220,221]). So far, four different G-patch co-factors of Prp43 have been identified: Ntr1/Spp382, Pxr1/Gno1, Pfa1/Sqs1, and Cmg1. They all share a domain rich in glycines, the G-patch, but have no homology outside this domain. These differences also result in different modes of interaction with Prp43 [222,223].

G-patch proteins interact with Prp43 and stimulate its ATPase and helicase activity via their G-patch [221,224–227]. Importantly, the different G-patch proteins are specific for different processes in which Prp43 functions. Ntr1 activates Prp43 in mRNA splicing, while Pxr1 and Pfa1 function in ribosome biogenesis [221]. The function of Cmg1 is so far only poorly characterized but seems to be unrelated to the so far described functions of Prp43 in ribosome biogenesis and mRNA splicing [225].

Prp43 is a component of multiple pre-ribosomal particles, as evidenced by the co-purification of 35S, 20S, and 27S pre-rRNAs, as well as assembly factors of 90S, pre-40S and pre-60S particles with Prp43 [228–230]. Depletion of Prp43, over-expression of helicase mutated variants, as well as various cold-sensitive *prp43* mutants all resulted in an accumulation of 35S pre-rRNA, accompanied by a reduction of 27S and 20S pre-rRNAs and consequently also of the mature rRNAs [228–230].

Apparently, Prp43 interacts with Pxr1 while bound to early pre-ribosomal particles, and with Pfa1 in the course of its function in later pre-ribosomal particles:

Pxr1 is a component of 90S and early pre-60S particles, and its deletion results in a defect in the early pre-rRNA processing steps at sites A_0_, A_1_ and A_2_ [224,226,231].

Pfa1 was also found to associate with multiple pre-ribosomal particles, but at later maturation stages than Pxr1. Upon Pfa1 affinity purification, mainly 20S pre-rRNA, along with 90S and pre-40S assembly factors is co-purified, suggesting that Pfa1 is a component of 20S pre-rRNA containing 90S particles and/or of pre-40S particles [229]. Additionally, also pre-60S assembly factors are co-purified with Pfa1, suggesting that in addition to SSU maturation, Pfa1 may also participate in LSU maturation [229].

The main binding sites of Prp43 on pre-ribosomal particles were identified by CRAC analyses as helix H44 of the 18S rRNA (Figure 1A) and helices H23/24, H29, H39/40 and H83 of the 25S rRNA (Figure 2A). Additionally, Prp43 was found to crosslink to multiple snoRNAs, preferentially of the box C/D type, with snR51 being represented much higher than all other snoRNAs, followed by snR72 and snR60 [232]. Notably, several of these snoRNAs have their rRNA binding sites in proximity to (although not overlapping with) the rRNA crosslinking sites of Prp43. snR51 base-pairs with 25S rRNA H86, which is in proximity to the Prp43 binding site in H83, while snR72 and snR60 bind in the area of helices H33 to H35 in the 25S rRNA, which are in proximity to the Prp43 binding site in H29 (Figure 2A).

Depletion of Prp43 causes the accumulation of snR72 and snR60 in pre-ribosome bound form [232]. In contrast, no such accumulation was observed for snR51, the snoRNA which was strongest enriched in the Prp43 CRAC experiments [232], suggesting, that Prp43’s interaction with this snoRNA may serve a structural role and not be relevant for the release of this snoRNA. However, also other snoRNAs with binding sites in proximity to Prp43 crosslinking sites (i.e. snR59, snR39, snR39b, and snR50) accumulated in pre-ribosome bound form upon Prp43 depletion, and also in an ATPase-dead *prp43* mutant, suggesting that Prp43 is required for the release of these snoRNAs [232]. Alternatively, the observed phenotypes could also be explained by a block in ribosome maturation that prevents the progression of the pre-ribosome maturation pathway, consequently preventing downstream events like the release of certain snoRNAs. Future studies will have to explore whether Prp43 has a direct function in snoRNA release.

A potential snoRNA-independent function of Prp43 is presumed for its binding site in H44 in the 3’ minor domain of the 18S rRNA (Figure 1A) [226,232,233]. It was observed that strains carrying a mutation in Prp43, or deleted for the non-essential G-patch cofactor Pfa1, showed a severe growth defect and a strong 20S pre-rRNA accumulation when additionally, the late pre-40S maturation factor Ltv1 was deleted or depleted [226,233]. As the endonuclease Nob1 acted as dosage suppressor for this defect, it is tempting to speculate that Prp43/Pfa1, together with Ltv1, function in an rRNA restructuring step that is the prerequisite for efficient 20S pre-rRNA processing by Nob1 [233]. This is further supported by the observation that in these mutants, also an aberrant 17S RNA arose due to aberrant 3’-5’ trimming of the 18S rRNA by the cytoplasmic exosome, removing helices H44 and H45 [226,233]. Hence, Prp43 may function in restructuring this region to ensure its stable, exosome activity resistant incorporation into pre-ribosomes along with positioning it in a way to allow for efficient cleavage by Nob1. Indeed, H44 of the 18S rRNA successively changes its orientation in the course of 90S and pre-40S maturation [22,23,33,87,90], and it is tempting to speculate that Prp43 participates in this restructuring. Notably, in addition to Prp43, also Dhr1 binds to H44 [102]. More studies will be necessary to unravel the mechanisms of H44 remodelling, including the potential role of Prp43 and Dhr1.

Despite these pieces of evidence towards potential targets, the exact molecular function of Prp43 in ribosome biogenesis still remains subject to speculation. In contrast, Prp43 is very well characterized on a structural level [170,171,219,234]. As typical for DEAH helicases, the two RecA domains together form the active site for ATP/binding and hydrolysis. The RNA binding channel lies at the intersection between this RecA core and the remaining three domains, i.e. the winged-helix (WH), helical bundle (HB) and oligosaccharide-binding (OB) domain. The single stranded RNA is believed to translocate through the RNA binding channel by moving one nucleotide per ATP hydrolysis round [170,219]. Recently, a partial structure of the human Prp43 ortholog DHX15 in complex with a G-patch protein (NKRF) was solved, providing more insight into the mechanism of activation of Prp43/DHX15 by G-patch proteins [235]. In that structure, the G-patch is positioned in an extended conformation at the back side of the RNA binding channel, and contacts two different domains, the WH domain and the RecA2 domain. The extended conformation provides sufficient flexibility to allow for RecA domain movements required for substrate processing. The mechanism of activation is believed to be by tethering the RecA2 and WH domains together via the brace-like G-patch domain, thereby keeping the RNA channel in a closed, RNA bound conformation, and consequently increasing processivity of the helicase [220,235].

### Has1 acts on both 90S and 60S precursor particles

Has1 is one of few helicases that was shown to be associated with both 90S and pre-60S assembly intermediates and thus plays a role in maturation of both ribosomal subunits [10,54–56,236]. The DEAD-box helicase has duplex destabilization activity and its RNA-stimulated ATPase activity is abolished in a dominant negative *has1* Walker A motif I mutant [237]. When Has1 is used as bait for affinity purifications it mainly co-purifies pre-60S assembly factors, together with some 90S factors that are, however, not retained at higher salt concentrations [238]. As polysome profiles of a temperature sensitive *has1* mutant reveal a 40S synthesis defect, whereas Has1 is predominantly found in 60S fractions, the helicase is expected to play an important role in 40S maturation despite an apparently transient association [239]. In line with this, *has1* mutants revealed an inhibition of A_0_, A_1_, and A_2_ cleavage resulting in accumulation of the 35S pre-rRNA but also some delay in processing 27S pre-rRNA species [239,240]. In addition, it was suggested that recruitment of the helicase to 90S and pre-60S ribosomes is mutually independent [238] and its catalytic activity could be required only for its 90S but not pre-60S function ([238]; V.M., manuscript in preparation).

#### Has1 potentially facilitates dismantling of the U14 snoRNA

Regarding the Has1 function in maturation of the small ribosomal subunit, depletion of Has1 as well as expression of *has1* catalytic mutants resulted in a release defect of the U14 snoRNA, which guides methylation of C414, from pre-ribosomes [191,241]. CRAC analyses revealed that wild type Has1 binds to the 18S rRNA at the 3‘ major domain around helices 31–41 as well as to H6 overlapping with the U14 binding site (Figure 1A) [64,238]. Further, it was suggested that Has1 interacts with both H13/H14 and the U14 snoRNA [64] and a crosslink site for a catalytic mutant was found at H17 and H18, which is also in close vicinity to the U14 binding region [238]. In conclusion, these data strongly indicate a function for Has1 in restructuring the U14 rRNA binding site and/or dismantling of the snoRNA from the 90S particle. In a recent preprint, an alternative role for Has1 was proposed, suggesting the helicase activity may trigger the dissociation of assembly factor Rrp36 from Rrp5 to allow its re-positioning from the body to the platform [242]. This would result in stabilization of an interaction between the UtpB components Utp13 and Utp22, promoting a switch in the UtpB subcomplex and the initiation of rRNA processing by A_0_ cleavage [242]. Since it was suggested that two or more copies of Has1 may be temporarily present at the same 90S particle it could be in principle feasible that Has1 would have also more than one function in 90S maturation [238].

#### Has1 acts on 25S rRNA domain I on early pre-60S intermediates and is released again before their translocation to the nucleoplasm

During pre-60S maturation Has1 is bound to a broad range of nucleolar maturation intermediates (Figure 3B). It was suggested that the helicase helps to re-arrange the 5.8S rRNA and 25S rRNA domain I by facilitating base pairing within H4 (established between a 5’ region of the 5.8S and 25S rRNA domain I). This Has1 activity is required for the stable Rpl17 incorporation and could later also play a downstream role in processing of the 27SB pre-rRNA species [239,240]. CRAC data (showing Has1 binding to 5.8S rRNA, ITS2, and helices H16, H17, H21, H22 of 25S rRNA domain I (Figure 2A)) and cryo-EM structures support a model in which Has1 could establish contacts between the 25S rRNA 5’ end and the 5.8S rRNA [54–56,64,238]. In several states of nucleolar pre-60S cryo-EM models, the helicase core of Has1 is (partly) visible at the top of the pre-60S foot structure in proximity to Rpl8, Rpl36, and assembly factors Nsa3, Nop15, and the C-terminus of Nop16 [54–56] (Figure 4B). Furthermore, it is in contact with the widely meandering N-terminal Erb1 tail, which together with its binding partner Ytm1 is released at the transition from state E to NE1 by the AAA^+^ ATPase Rea1 [53,54,243]. Since Has1 is also not present on state NE1 particles anymore it might dissociate together with the Erb1**–**Ytm1 complex during this remodelling step. The cryo-EM structure of Has1 bound to pre-60S particles also reveals that H16 of 25S rRNA domain I is clamped between the two Has1 RecA-like domains [56] (Figure 4B; H16 is shown in purple, the crosslink site at H21/H22 that could interact with the short C-terminal Has1 extension is shown in orange). However, since H16 is not accommodated in a typical position for DEAD-box helicase substrates and the RecA-like domains are in an open inactive conformation, it was suggested that H16 rather serves as docking site than as unwinding substrate [56,165]. Moreover, the ATP-binding pocket of pre-60S bound Has1 was empty [56], which is in agreement with the finding that the release of Has1 from state E particles is not dependent on the catalytic activity of the helicase but that it is pulled off as result of the Rea1-dependent restructuring step [56] (V.M., manuscript in preparation). Thus, in contrast to its 90S function, the role of Has1 in pre-60S maturation could be ATP-independent and may only require its physical presence.

### Mtr4 unwinds pre-rRNA spacer elements on 90S and pre-60S particles for degradation by the nuclear exosome

**Figure 5.**
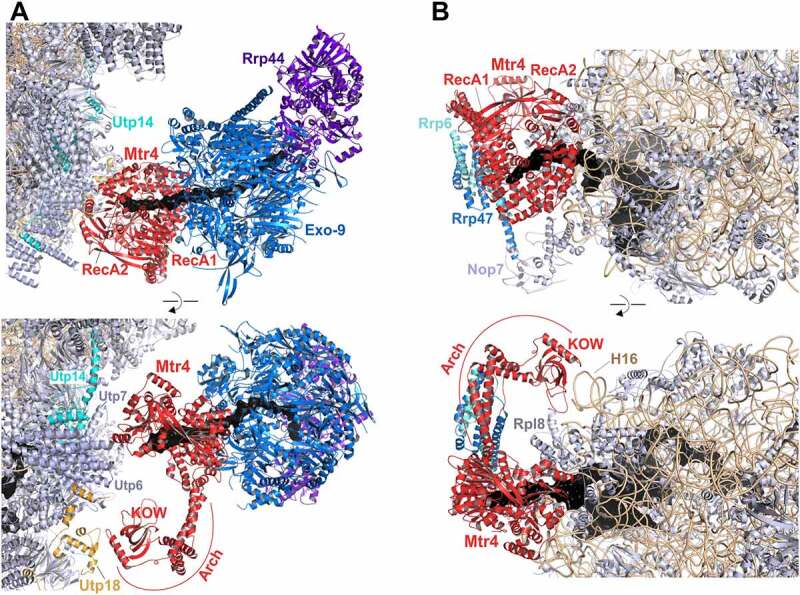
Cryo-EM structures of Mtr4 on 90S and pre-60S intermediates channelling its substrate RNAs for exosomal degradation.

Mtr4 is another helicase that is acting on both 90S and pre-60S maturation intermediates. As a processive helicase it shows ATPase and 3‘-5‘ RNA unwinding activity that is stimulated by structured RNA species such as tRNA in vitro [244]. It is a member of the Ski2-like family of helicases and, in contrast to its cytoplasmic counterpart Ski2, it is solely associated with the nuclear exosome targeting a wide range of RNA species, including mRNAs, snoRNAs, and ncRNAs, for decay and surveillance mostly within a Trf4**–**Air2**–**Mtr4 polyadenylation (TRAMP) complex [245–251]. In contrast, its functions in the productive ribosome assembly pathway are TRAMP complex independent. As exosome-associated helicase on pre-ribosomes, Mtr4 unwinds the 5’ ETS and ITS2 for subsequent exosomal processing on 90S and pre-60S particles, respectively [72,86,101,245,252–254] (Figure 5). In addition to the helicase core, Mtr4 possesses an N-terminal ß-hairpin domain and an insertion within its C-terminal domain called arch, followed by a helical bundle that is placed on a cleft between the two RecA-like domains in Mtr4 crystal structures [255,256]. The arch insertion, which is conserved between Mtr4 and Ski2, is composed of a coiled-coil stalk and a globular ß-barrel termed KOW (Kyrpides-Ouzounis-Woese) domain, which is also found in r-proteins and has RNA-binding activity [256,257]. Mtr4 is recruited to both its 90S and pre-60S pre-ribosomal substrates through two specific adapter proteins, Utp18 and Nop53, respectively, which bind to the arch via an arch interacting motif (AIM) [254]. Notably, the AIM-interacting residues of the Mtr4 KOW domain are not present in the cytoplasmic counterpart Ski2, providing specificity to Mtr4 for its nuclear functions. Once bound to its pre-ribosomal substrate, Mtr4 recruits the core exosome Exo-9 (consisting of nine protein components) forming a barrel-like cage to which the two processive nucleases Rrp44 and Rrp6 (only for the nuclear exosome) dock [258–260]. Moreover, recent data suggest that Rrp6 (human EXOSC10) establishes a second physical connection between the exosome and the 90S particle [18,261]. Upon substrate RNA unwinding by the Mtr4 helicase core, a now single stranded RNA can get channelled through the exosome core for exonucleolytic 3‘-5‘ degradation [72,86,101].

Mtr4 (red) is associated via its arch domain with 90S (A) and pre-60S (B) intermediates (PDB: 6LQS and 6FT6, respectively). (A) The A_0_-cleaved 5’ ETS RNA fragment (black) is channelled through the helicase core to the nuclear exosome core (blue) docked to exonuclease Rrp44 (violet). While the Mtr4 RecA-like domains contact Utp6, the arch domain is close to an N-terminal part of Utp18 (orange). Note that the AIM-containing region within the Utp18 N-terminus is not resolved in the structure. Utp14 is indicated in cyan. (B) The 3’ extension of the 5.8S rRNA (black) is channelled through the helicase core, while the arch domain is interacting with 25S rRNA H16 and r-protein Rpl8. The exosome components Rrp6 and Rrp47 are depicted in cyan and light blue, respectively. Note that AIM-containing Nop53 is not visible in the structure.

#### Mtr4 dislodges the 5’ ETS from 90S particles

Mtr4 binds to 90S particles already at a step prior to A_1_ cleavage and is released again upon A_1_ cleavage and subsequent 5’ ETS degradation [19,86,101] (Figure 3A). It was suggested that Mtr4 dislodges the 5’ ETS in a sequential manner with the initial 5’ ETS remodelling potentially required as a pre-requisite for A_1_ cleavage. After A_0_ cleavage and upon exosome recruitment via Mtr4, the 5’ ETS-A_0_ fragment might be, starting with the 3’ end, channelled through the helicase core into the exosome cavity. On recent cryo-EM structures, the helicase is resolved on 90S particles in proximity to the 3’ end of the 5’ ETS at the base of dislodged H9 at a composite surface formed by Utp6, the Sof1 module (Sof1**–**Utp7**–**Utp14), Fcf2, Utp14, and the AIM-containing Utp18 N-terminus [86,101], (Figure 5A). Whereas the N-terminal Utp18 AIM is not visible in the available 90S cryo-EM structures, a visible part of the N-terminus is positioned close to the Mtr4 KOW domain, which would allow for a direct Utp18**–**Mtr4 interaction. Mtr4 binding may potentially be stabilized also by α-helical elements of Utp14, a co-factor of the helicase Dhr1 [177].

#### Mtr4 unwinds the 3’ end of the 7S pre-rRNA on nucleoplasmic 60S precursors

Mtr4 is recruited to the pre-60S particle, by the adaptor Nop53, which binds during the translocation of the LSU precursor from the nucleolus to the nucleoplasm initiated by the AAA^+^ ATPase Rea1 [53,63,243] (Figure 3B). Together with assembly factors Nsa3, Nop15, Rlp7, and Nop7 as well as the ITS2 RNA, Nop53 is part of the pre-60S foot structure [61]. Following endonucleolytic C_2_ cleavage within ITS2 of the 27SB pre-rRNA, Mtr4 acts on the 3’ end of the resulting 7S pre-rRNA, feeding it to the attached exosome [65,66,72] (Figure 5B). This Mtr4 maturation step occurs around a stage of Rea1 re-binding to facilitate final construction of the L1 stalk (formed by 25S rRNA domain V), and re-arrangement at the CP including a crucial 180° rotation of the 5S RNP, which together finally produces export-competent pre-60S subunits [53,59,61,70,74,262]. Using a reconstitution approach with Nop53-derived pre-ribosomal intermediates and recombinantly added exosome components, it was possible to visualize Mtr4 attached to the residual pre-60S foot structure, with the arch’s KOW domain contacting helices H15/H16 of 25S rRNA domain I and Rpl8, and the helicase core positioned in proximity to H76 and H79 of domain V [72] (Figure 5B). Whereas the bait protein Nop53 that interacts with the KOW domain as well as other assembly factors of the foot are not resolved in the cryo-EM structure, Nop7 is the only remaining foot-factor at this stage of processing and might contribute to exosome docking via an interaction with a long protruding α-helix of the exosome component Rrp47 [72] (Figure 5B). The 3’ extension of the 5.8S rRNA is channelled through the Mtr4 helicase catalytic core for degradation by the exosome nucleases Rrp44 (5.8S+30 processing) and subsequently Rrp6 (6S processing) [65,66,72]. In human cells, Mtr4 can be also recruited by different arch-interacting adapter proteins [263]. Interestingly, NVL2, the homolog of the AAA^+^ ATPase Rix7 that acts on earlier pre-60S intermediates, is among those interacting proteins, suggesting that in higher eukaryotes, Mtr4 might have additional functions in 60S maturation or in clearance pathways targeting aberrant pre-ribosomal particles for degradation.

## RNA helicases in 60S particle maturation

### Dbp6, Dbp7 and Dbp9 mediate rRNA folding and snoRNA release in early pre-60S particles

Dbp6, Dbp7 and Dbp9 are functionally connected DEAD-box helicases. Dbp9 exhibits ATPase activity and RNA as well as DNA helicase activity in vitro [264], and Dbp7 was shown to display RNA-dependent ATPase activity [142,265]. For Dbp6, catalytic activity has not been experimentally demonstrated yet, however the observation that helicase motif I mutants are inviable and confer a dominant-negative growth phenotype [266] suggest that catalytic activity is important for Dbp6’s function.

The genes encoding these helicases are part of a common genetic network additionally including the genes encoding the ribosome assembly factors Npa1 (also known as Urb1), Npa2 (also known as Urb2), Nop8, and Rsa3, as well as the r-protein Rpl3 [267–271]. Importantly, Dbp6 also forms a physical complex with Npa1, Npa2, Nop8, and Rsa3, the Npa1 complex [52,57]. Within that complex, which also forms when rRNA transcription is inhibited, Dbp6 interacts with both Npa1 and Npa2, two large structural components of the complex [52]. It is up to now not clear whether Dbp7 is in direct physical contact to that complex. It is however noteworthy that Dbp7 co-purifies, beside other pre-60S maturation factors, significant amounts of all Npa1 complex members, including Dbp6 [265]. Moreover, sucrose gradient fractionation of Npa2-TAP purifications yielded, in addition to particles sedimenting at the size of pre-60S, also small complexes containing Npa1, Dbp6, Nop8 and Rsa3, but also Dbp7 and Dbp9 [271]. Notably, the Npa1 core complex was isolated under stringent high-salt conditions [52]. It is tempting to speculate that Dbp7 and Dbp9 are also part of the Npa1 complex but that their interaction with the complex is more transient than of the core members and therefore salt sensitive.

Compared to Dbp6 and Dbp7, less information is available about Dbp9. *DBP9* can act as a dosage suppressor of some *dbp6* mutations, suggesting that Dbp6 and Dbp9 have partially overlapping functions, and that in the case of not fully functional Dbp6, Dbp9 can at least partially take over Dbp6’s functions [183].

Dbp6 and Dbp9 depletion, as well as the *dbp7-1* mutation all lead to similar rRNA processing defects, including the accumulation of 35S and 32S pre-rRNA and consequently a reduction of 27SA_2_ pre-rRNA and later precursors in the 60S maturation pathway [183,267,269]. However, in the case of Dbp7, it was shown that short-time depletion actually leads to an accumulation of 27SA_2_ pre-rRNA, suggesting that the primary effect of absence of Dbp7 is a delay in maturation of 27SA_2_ pre-rRNA to subsequent species [265].

At what maturation stage are the Npa1 complex and Dbp6, Dbp7 and Dbp9 bound to pre-ribosomal particles? Both Dbp6 and Dbp7 co-purify predominantly 27SA_2_ pre-rRNA. Additionally, small amounts of 35S and 32S pre-rRNAs co-purify with both proteins, suggesting that they additionally associate with 90S particles and that they are present during the 90S to pre-60S transition [52,265]. Last but not least, also small amounts of 27SB pre-rRNA co-purify both with Dbp6 and Dbp7, suggesting that both proteins dissociate from pre-60S particles soon after 27SA_2_ pre-rRNA has been converted to later forms [52,265]. A similar analysis has not been performed for Dbp9. Interestingly, comparison of the proteins present in early pre-60S particles purified via Rrp5 and the intermediate pre-60S particles purified via Nsa2 revealed high levels of all three helicases in the Rrp5-purified particles, while only Dbp7 and Dbp9 were present at low amounts also in the Nsa2-TAP particle [108]. These data, together with unpublished data from the Pertschy lab, suggest that Dbp7 and Dbp9 dissociate from pre-60S particles slightly later than Dbp6.

So, what is the actual function of these helicases within early pre-60S particles? Generally, the Npa1 complex is believed to act as a chaperone in early rRNA folding [52]. The large (~200 kDa) backbone protein Npa1 forms contacts with rRNA elements in 25S rRNA domains I, II and VI according to crosslinking analyses (Figure 2A) [52]. Remarkably, Npa1 also interacts with snR190, which base pairs to rRNA domains I and V in the 25S rRNA, in proximity to the Npa1 binding sites. Based on that, both Npa1 and snR190 are believed to bridge distant rRNA regions and thereby promote rRNA folding and compaction [52,142]. Importantly, the same rRNA elements interact with Rpl3 in mature 60S ribosomes, hence the Npa1 complex and snR190 may ensure that these distant 25S rRNA domains are pre-arranged to allow for efficient Rpl3 binding. Dbp6 has been suggested to play an important role in this chaperone activity [52].

Dbp7 crosslinks to H89 and H90 in domain V, and to H94 and H98 in domain VI of the 25S rRNA [265] (Figure 2A). The H94 and H98 crosslinking sites are adjacent to or overlapping with, respectively, two crosslinking sites of Npa1 (Figure 2A) [142], suggesting that Dbp7 might be bound to Npa1 while acting on this RNA region. The domain V crosslink is in proximity to the snR190 binding site and moreover, Dbp7 also crosslinks to snR190 [265]. Several lines of evidence suggest that Dbp7 is responsible for release of snR190: (1) *DBP7* is not essential, however its deletion results in a strong growth defect. This phenotype can be alleviated by mutations in *SNR190* predicted to reduce base-pairing with its rRNA binding sites, but also by mutations in 25S rRNA H73 of domain V base-pairing with snR190 [142]. Hence, weakening the binding of snR190 to rRNA can partially compensate for the loss of Dbp7. (2) snR190 is retained in pre-60S particles in the absence of Dbp7 [142,265].

Apart from this strong functional connection to snR190, Dbp7 binds also to snoRNAs snR10, snR42, snR5, and snR61, which all base-pair with rRNA elements in proximity to Npa1’s rRNA binding sites. It is up to now unclear whether Dbp7 is also involved in release of some of these snoRNAs [265].

### Dbp3 may contribute to snoRNA recycling and efficient 27SA_3_ processing

The non-essential DEAD-box protein Dbp3 shows ATPase activity stimulated by RNA and unwinds short RNA duplexes in vitro [188,189,272]. Depletion of the protein results in a 60S biogenesis defect (as obvious from polysome profiles) and increase of the 35S, 33/32S, 27SA_2_, and most prominent the 27SA_3_ pre-rRNA species, which could indicate a role of Dbp3 in facilitating A_3_ cleavage by the RNase MRP [272–274]. As *DBP3* is not essential, with a *dbp3*Δ strain showing a cold-sensitive slow-growth phenotype, and the lack of Dbp3 or expression of a catalytically inactive mutant did not clearly impair production of the mature 25S rRNA, only a minor contribution of this helicase to rRNA maturation was assumed [272,274]. Nevertheless, in absence of Dbp3 several snoRNAs (i.e. U18, U24, snR39, snR39b, snR50, snR55, snR59, snR60, snR61, snR67, snR69, snR74, snR79) accumulated on pre-ribosomes, going along with a reduced global extent of 2′-O-methylation at various sites within the 25S rRNA but mainly clustered around the PTC or tRNA binding sites [272]. Dbp3 may promote an efficient recycling of the trapped snoRNAs ensuring their sufficient availability for stoichiometric 2′-O-methylation on all precursor particles. Interestingly, depletion of the C/D box snoRNP component Nop56 rescued the pre-rRNA processing defect observed in *dbp3*Δ cells, potentially by bypassing the requirement of the helicase for snoRNA recycling [272]. Dbp3 was found associated with very early Npa1-derived 60S precursor particles [57]; thus, since the considered 2′-O-methylations occur at early pre-60S maturation stages, a role of the helicase in snoRNA release from these precursors is feasible. However, Dbp3’s binding site on the pre-ribosome, as well as its restructuring target remain unknown, and its activity may alternatively/additionally also contribute to folding of ITS1 and recruitment of the RNase MRP for A_3_ cleavage.

### Drs1 is required for co-transcriptional A_2_ cleavage and downstream 60S biogenesis steps

The DEAD-box helicase Drs1 is associated with early to intermediate nucleolar 60S precursor particles (e.g. Npa1-, Nop7-, Nsa1-, Ytm1-derived intermediates) and is released again before late nucleolar (i.e. Ytm1 E80A particles, state E) maturation steps [54,57,67,275] (Figure 3B). When pre-ribosomes are purified directly via Drs1 as bait protein, nucleolar pre-60S particles are obtained, containing pre-60S assembly factors like Nop7, Nsa1, Mak16, Ebp2, Rpf2, Nog1, and helicase Dbp10, whereas the later joining Rrp17 and helicase Spb4 (see below) are absent (V.M., unpublished data).

In cells harbouring a cold-sensitive *drs1* mutant that was still capable of pre-60S binding, 25S rRNA synthesis was impaired and the 27SB pre-rRNA accumulated [276,277]. Distinct cold-sensitive *drs1* alleles (with mutations within the catalytic RecA-like domains) further showed synthetic lethality when combined with mutant alleles of the gene encoding the foot-factor Nop7 [276]. Moreover, Drs1 shifted into a ribosome-free Nop7 subcomplex upon shutdown of *de novo* rRNA synthesis [278] and interacted with the Nop7**–**Erb1**–**Ytm1 subcomplex in vitro [275]. In line with this, dissociation of Nop7**–**Erb1**–**Ytm1 from pre-60S particles lacking Drs1 during sucrose gradient centrifugation indicates that the helicase might be required for stable incorporation of this module (V.M., unpublished data). In addition to that, Nsa1 affinity-purifications upon Drs1 depletion revealed a massive amount of free bait protein, suggesting a (possibly indirect) role of Drs1 for Nsa1 assembly as well (V.M., unpublished data).

In contrast to the 27SB pre-rRNA accumulation in cold-sensitive *drs1* mutants that were capable of pre-60S binding, depletion of Drs1 leads to accumulation of 27SA_2_ and 27SA_3_ and, notably, 35S pre-rRNAs [275]. Furthermore, very early pre-60S assembly factors as well as r-proteins of the small subunit were enriched on pre-60S particles purified via early-binding Nop7 upon Drs1 depletion. Thus, in complete absence of the helicase clearly an earlier maturation step is impaired than in the mutant situation and based on the accumulation of 35S pre-rRNA (which does not arise when co-transcriptional processing occurs), it was proposed that co-transcriptional A_2_ cleavage could shift more to a post-transcriptional ITS1 processing [275]. Semiquantitative mass spectrometry analyses, revealing enormous accumulation of early pre-60S (e.g. helicase Dbp6, Npa1, Rrp5, Noc1, Mak5, Nop4, Nop12), as well as a plethora of 90S assembly factors (including Dbp4 and factors of the UTP-A, UTP-B, UTP-C, Kre33 modules) and a strongly decreased association of later pre-60S factors (e.g. Rlp24, Tif6, Noc3, Nsa2, Nog2) on Drs1-depleted Nop7 particles, support such a function in early ribosome assembly [275] (V.M., unpublished data).

Taken together, the physical presence of Drs1 could be required at an early maturation stage to allow A_2_ cleavage and separation of the first already assembled 60S factors, while its catalytic function might promote later 60S assembly steps prior to 27SB processing. Potential folding targets may be in vicinity of the Nop7 and Erb1 binding site within 25S rRNA domain III for the late Drs1 function, whereas the binding site for its early function might be different. A similar dual role during ribosome assembly can be assumed for the human Drs1 homolog DDX27, as it binds with its N-terminal extension to the human Nop7**–**Erb1**–**Ytm1 (Pes1**–**Bop1**–**WDR12) module and, independent of this interaction, contributes to processing of the 47S pre-rRNA [279].

### Mak5 facilitates early pre-60S maturation and is associated with 25S rRNA domain II

A point mutation within the ATP-binding Walker A motif I of Mak5 causes deficiency in maintenance of the M1 dsRNA virus yielding a killer minus phenotype [280]. Further, the DEAD-box helicase is essential for maturation of the 60S subunit and its depletion results in a 27SA to 27SB processing defect [64,280]. Its ATPase activity is stimulated in presence of RNA and abolished in DEAD (Walker B/motif II) or SAT (motif III) mutants [64]. Mak5 has both N- and C-terminal extensions of similar size flanking the helicase core, of which only the conserved C-terminus is essential for its function [281]. The helicase is genetically linked to assembly factors Ebp2, Nop16, Rpf1, Rpl14, and, remarkably, a nonsense mutation within the C-terminal extension bypasses the requirement for the otherwise essential assembly factor Nsa1 [281]. Mak5 only transiently or weakly interacts with early 60S precursors purified via Npa1, Ssf1, Nsa1, or Nop7 [57,281] and CRAC analyses revealed Mak5 binding to 25S rRNA domain II helices H35a to H40 [64]. The identified Mak5 binding sites overlap with base-pairing sites of several snoRNAs (i.e. snR5, snR8, snR51, snR60, snR82), however, depletion of Mak5 did not significantly change snoRNA levels on pre-ribosomal complexes, suggesting the helicase is not involved in the association or dissociation of these snoRNAs [64]. Footprinting analyses indicate that RNA bases within the crosslink site at H39 are accessible for DMS-modification in pre-ribosomal particles from wild-type but not from Mak5 depleted cells. Based on these structural differences in H39 in the absence of Mak5, it was proposed that Mak5 facilitates restructuring around this area on intermediate nucleolar 60S precursors [64]. Whereas the identified Mak5 binding site is not in direct proximity to most of the functionally interacting ‘Mak5 cluster factors’, the crosslink site is in contact with a long kinked Ebp2 α-helix within the essential Ebp2 core, which becomes visible only on pre-60S cryo-EM structures at maturation state D/E [54,281]. Notably, a mutation introducing a stop codon at the beginning of this Ebp2 α-helix not only bypasses the requirement for Nsa1 but also shows synthetic lethality combined with the *mak5* G218D (motif I) allele [281]. This suggests that Ebp2 could contribute to a potential Mak5 re-arrangement of the rRNA region around H36/37 and H39.

### Dbp2 might help in proximal stem formation during 60S assembly

Dbp2 is a DEAD-box helicase that has several non-essential cellular functions including its contribution to ribosome assembly [282–286]. Both Dbp2 and its human homolog DDX5 display ATPase and RNA-unwinding activity, which for the human variant is strongly increased by a mammalian-specific C-terminal extension [287,288]. In *dbp2*Δ cells and less pronounced in catalytic Walker A motif I mutants, the levels of both mature 25S and 18S rRNA are decreased and the 35S and to some degree the 27S pre-rRNAs accumulate, whereas polysome profiles possibly indicate a slight 60S synthesis defect [282]. Mass spectrometry analyses detected Dbp2 on early to intermediate nuclear pre-60S particles (i.e. Nsa3, Nog1, Nug1 particles) (Figure 3B), however, its association is only transient or very unstable [236,289]. Interestingly, Dbp2 becomes enriched on pre-60S preparations isolated from catalytic mutants of the DEAD-box helicase Dbp10, suggesting the requirement of Dbp10 activity for Dbp2 dissociation (V.M., unpublished data).

While the role of yeast Dbp2 in ribosome biogenesis is poorly characterized, there are data available for its human homologs DDX5 and DDX17 that fulfil a redundant function for cell proliferation and viability and, at least in case of DDX5, can complement the 60S biogenesis defect of *dbp2*Δ cells [282,290]. A 32S pre-rRNA (corresponding to yeast 27S pre-rRNA) processing defect observed upon DDX17 knockdown is less pronounced for DDX5. Simultaneous knockdown of DDX5 and DDX17 results in increased cellular levels of the metazoan-specific U8 C/D box snoRNA and its dissociation from pre-ribosomes is impaired under this condition [290]. Studies in *Xenopus* oocytes showed that U8 base-pairing with the 5’ end of the 28S pre-rRNA is required for 28S and 5.8S production [291,292]. It was suggested that DDX5/DDX17 might help to dislodge the snoRNA from the 28S rRNA domain I, thereby promoting formation of the proximal stem (by annealing of the 5.8S 3’ end to the 28S 5’ end) [290,291]. A similar possibly redundant function for U8 displacement was described also for the mammalian DEAD-box helicase DDX51 [293]. While there is no functional homolog of the U8 snoRNA in yeast, Dbp2 might still contribute to the formation of the proximal stem, which is required for efficient rRNA processing also in yeast [294]. The helicase may play a role in re-arranging an ITS2 hairpin stem, which in yeast was proposed to provide the vertebrate U8 function in cis to prevent a premature folding of the proximal stem early during nucleolar 60S assembly [295]. As a role in promoting base-pairing between the 5.8S and 25S rRNA domain I was also suggested for Has1 (see above), Dbp2 and Has1 might have complementary or possibly redundant functions in early pre-60S maturation.

### Dbp10 restructures rRNA within the immature PTC promoting GTPase Nug1 and methyltransferase Spb1 recruitment

The essential DEAD-box helicase Dbp10 is required for processing of the 27SB to the 25.5S and 7S pre-rRNA species [296] and interacts both genetically and physically with the circularly permuted GTPase Nug1 [69,297]. According to a hierarchical recruitment model of assembly factors and r-proteins needed for 27SB maturation (‘B-factors’), Dbp10 is among the earlier of these factors preceding the binding of Nog1, Nsa2, and Nog2 to allow for endonucleolytic cleavage at site C_2_ [298–301]. The helicase is enriched with intermediate nucleolar Nsa3-, Nug1-, Nsa1/Ytm1- (structural states A-C) particles but not with the succeeding Ytm1 E80A (state E) derived particles [54,236,297] (Figure 3B). CRAC analyses revealed that Dbp10 and Nug1 have proximal and overlapping binding sites on the 60S intersubunit side at 25S rRNA domain V around the evolving PTC [69]. In addition to its domain V crosslink at helices H89-H92, an additional Dbp10 crosslink in H61 and H63/H64 within 25S domain IV was found that could represent an interaction site for the long C-terminal Dbp10 auxiliary domain. In contrast, H89-H92 were proposed as target for the catalytic domain facilitating re-arrangements around the PTC rRNA [69]. Exactly this rRNA region becomes folded and structurally visible at the transition from structural state B to state C (Figures 2B and 3B), suggesting that the helicase restructures H89-H92 during this maturation stage [54]. Of note, this Dbp10 rRNA binding site partially overlaps with a recently identified binding site of the earlier-acting Dbp7 [265] (Figure 2A; see above); hence, the two RNA helicases might sequentially act on this region, Dbp7 by releasing snR190, and Dbp10 by performing subsequent restructuring events.

DbpA, the bacterial homolog of Dbp10 in *E. coli*, binds as well to the equivalent rRNA region around the PTC. It was suggested that DbpA binds with its C-terminal RNA recognition motif (RRM) to 23S rRNA H92 [160,302–304], which results in a tight interaction between the RRM and the RecA2 domain, inducing the closed helicase state and triggering RNA unwinding [305]. The direct restructuring target of DbpA was suggested to be a single-stranded region between H89 and H90 [305,306].

Assuming a similar role of Dbp10 in conformational restructuring of the immature PTC in eukaryotes, its activity could be important for the access of the two methyltransferases Nop2 and Spb1 to modify their target nucleotides C2870 (at the base of H89) and G2922 (in H92), respectively [307,308]. In affinity purifications with Dbp10 as bait, both methyltransferases are enriched revealing they can be present on the same pre-60S particles together with the helicase (V.M., unpublished data). However, while upon depletion of Dbp10 or in dominant catalytic *dbp10* mutants (which efficiently associate with pre-60S particles), Nop2 is still recruited, Spb1 fails to efficiently bind to pre-60S particles. Interestingly, also the recruitment of the GTPase Nug1 is fully abolished in these *dbp10* mutants (V.M., unpublished data), which is surprising as vice versa Dbp10 was also not efficiently assembled upon Nug1 depletion [69]. Furthermore, affinity purifications of pre-60S particles using several catalytic Dbp10 mutants directly as bait protein, revealed a specific failure of Nug1 to bind to such intermediates, whereas the pattern of the other co-purified assembly factors remained nearly unchanged compared to wild-type Dbp10 purifications (V.M., unpublished data). The C-terminal auxiliary domain of Dbp10 has an essential eukaryote-specific extension that mediates an interaction with both the methyltransferase Spb1 and an N-terminal Nug1 α-helix, which is the only visible part of Nug1 in available cryo-EM structures [54,61] (V.M., unpublished data). Furthermore, Spb1 also interacts with and has overlapping binding sites with Dbp10 on this N-terminal Nug1 α-helix, suggesting a sequential association of first Dbp10 and, potentially after Dbp10-dependent restructuring, next Spb1 with the Nug1 N-terminus (V.M., unpublished data).

Taken together, Dbp10 activity promotes the recruitment of its interaction partners Nug1 and Spb1. Re-arrangement of the PTC (domain V, H89-H92) could make this region accessible for Spb1- and potentially Nop2-mediated base methylation. However, it remains unclear at which exact maturation stage the two methyltransferases are triggered to modify their rRNA targets.

### Spb4 promotes re-arrangements within 25S rRNA domain IV at a late nucleolar maturation stage

The DEAD-box helicase Spb4 is essential for cell viability and for biogenesis of the 60S subunit and is mainly bound to 27SB pre-rRNA containing pre-60S particles [309,310]. Upon its depletion and in *spb4* catalytic mutant backgrounds, processing of the 27SB precursor is blocked [64,309,310] and, in line with this, Spb4 depletion impairs pre-60S recruitment of the late B-factor Nog2 [301]. More specifically, Sbp4 depletion arrests pre-60S maturation at a stage directly prior to Nop53 and Rrp17 joining, and Ytm1 release (V.M., manuscript in preparation). Accordingly, Spb4 affinity purifications display a protein pattern highly similar to Ytm1 E80A-derived particles impaired for Ytm1**–**Erb1 removal by the AAA^+^ ATPase Rea1, corresponding to structural state E [54]. Such particles are enriched for assembly factors typically found on late nucleolar pre-60S particles (e.g. Spb1, Noc3) and contain stoichiometric amounts of Spb4’s binding partner Rrp17 (V.M., manuscript in preparation). As the helicase is not present on the subsequent state NE1 intermediates [53], it acts on a very narrow range of late nucleolar pre-60S precursors (Figure 3B). Spb4 binds to such intermediates at a hinge region at the base of a highly flexible arm formed by 25S rRNA domain IV (crosslink site at H62/H63/ES27) (Figure 2A), which later also anchors the pre-60S export factor Arx1 [54,55,64]. As footprinting analyses indicated that the rRNA around H62/H63 was also less accessible for DMS-mediated modification in cells depleted for Spb4, this region could indeed represent the restructuring target of the helicase [64]. In agreement with this assumption, H62 is not visible in the state E cryo-EM structure but becomes structured and visible in subsequent NE1 particles after Spb4 dissociation [53,54].

A second major crosslink site was found at H99/H100/H101 (25S rRNA domain VI) (Figure 2A), which was proposed to act as docking site for the Spb4 C-terminal extension rather than a target for remodelling [64]. Truncation constructs of the C-terminal Spb4 auxiliary domain indeed did not efficiently co-purify pre-ribosomal particles (V.M., manuscript in preparation), which was shown also for the human Spb4 homolog DDX55 [311]. Notably, likewise to C-terminal truncation constructs, affinity purifications using ATP-binding or hydrolysis deficient Spb4 mutants as bait proteins revealed strongly decreased co-enrichment of pre-ribosomal particles, which indicates that both ATP binding and hydrolysis by the helicase could be required already at the stage of its initial association with the pre-ribosome (V.M., manuscript in preparation). Thus, the Spb4 rRNA re-arrangement step might simultaneously go along with the pre-60S binding step requiring its catalytic activity, which could potentially apply also for other DEAD-box helicases.

## Concluding remarks

In the course of ribosome biogenesis, pre-rRNAs are transcribed by RNA polymerase I and III and successively fold into the tremendously complex three-dimensional rRNA structure of mature ribosomes. rRNA folding comprises numerous individual (and sometimes interdependent) folding events, for the majority of which the exact choreography and mechanisms remain elusive. RNA helicases are believed to actively mediate such folding and unfolding events in ribosome biogenesis. However, it has to be acknowledged that due to the sheer multitude of structural changes occurring during this process, the only 19 RNA helicases participating in ribosome biogenesis in yeast can only be responsible for a minority of these events, while most restructuring events can likely proceed without the help of enzymes. Nevertheless, the fact that many RNA helicases are known to act on particularly important or complex RNA folds, like the CPK or the PTC, suggests that RNA helicases promote key restructuring events in ribosome biogenesis.

In line with the observation that most rRNA folding events occur in the very early steps of ribosome biogenesis, almost all RNA helicases in ribosome assembly function in the nucleolus.

Although the naive idea that RNA helicases function in directly unwinding RNA duplexes may be correct in several instances, and putative functions in such RNA remodelling events have been proposed for many RNA helicases in ribosome biogenesis, there are only few examples of RNA helicases for which the precise substrate and function is definitely proven. The prime example for a well-understood helicase is Dhr1, for which ample biochemical, genetic and structural data demonstrate the function in release of the large scaffold snoRNA U3 from the 18S rRNA. In turn, the liberated single-stranded rRNA elements can undergo interactions with their cognate hybridization partner sequences, leading to the formation of a central structural and functional element of the 18S rRNA, the CPK. As the CPK is a composite structure formed by rRNA elements which are more than 600 nucleotides apart, it is not surprising that coordination and enzymatic assistance is required to establish this complex fold.

A function in snoRNA release was also found for Dbp7 and proposed for Dbp4, Rok1, Prp43, Has1, Dbp7, Dbp3, and Dbp2, and it is tempting to speculate that in all these cases, the release of the respective snoRNAs and the resulting liberation of rRNA elements triggers strategically important rRNA folding events. Of note, all the snoRNAs that are known to have mainly structural roles in ribosome biogenesis are believed to be released by RNA helicases, suggesting that in contrast to the more transiently binding modification-guiding snoRNAs, more effort is needed to remove the snoRNAs with chaperone function.

Other RNA helicases have been postulated to directly act on rRNA helices and mediate their restructuring, i.e. Mak5, Dbp10, Spb4, and Prp43. However, in lack of molecular mechanisms, detailed insights into such restructuring processes remain mainly elusive. Last but not least, at least one RNA helicase, Mrt4 does not target rRNA but spacer elements, and the unfolding mediated by this RNA helicase does not serve the goal to eventually generate a new rRNA fold, but instead makes these spacer elements susceptible to exonucleolytic degradation.

Despite common enzymatic mechanisms, the functions and exact sites of action of RNA helicases in ribosome biogenesis are diverse. All of them share the domain architecture typical for DEAH/DEAD box helicases, however, many of them carry N- and/or C-terminal extensions specific to the respective proteins, which may be involved in determining the helicase binding sites and different unique functions. Additionally, some of them have specific co-factors ensuring that the helicases become active at the correct time. Many helicases associate with pre-ribosomal particles only transiently and dissociate again after having performed their functions, while some of them are more stably bound to pre-ribosomal particles and could even be visualized in a pre-ribosome bound stage by cryo-EM. These likely have, in addition to their catalytic function, also a non-catalytic, structural function in ribosome biogenesis.

Coming back to the title of this article and reconsidering the role of the function of RNA helicases in RNA folding, it remains unknown how many RNA helicases directly mediate rRNA folding. Such direct rRNA folding activity and promotion of rRNA duplex formation may be feasible for helicases that possess RNA strand annealing activity (e.g. Rok1). In contrast, many RNA helicases appear to promote rRNA folding indirectly by removing ‘folding inhibitors’ like snoRNAs in the case of processive RNA helicases (DEAH and Ski2-like family) or local melting of rRNA–snoRNA base pairs in case of DEAD-box helicases. These actions then likely promote the folding of the now liberated rRNA elements. In addition to snoRNAs, also proteins can act as folding inhibitors, and it is conceivable that helicases might also be engaged in the release of proteins from rRNA, as has been postulated for example in the case of Rok1 (Rrp5 release) and Has1 (Rrp36 release).

As detailed in this article, the definite function of most helicases is not yet clear and remains subject to speculation, and in several instances, conflicting data exist on the putative function of a helicase. Moreover, some RNA helicases have overlapping binding sites, and it is unclear how their actions are coordinated. Some helicases even remain almost uninvestigated. Major challenges in studying RNA helicases are the often-transient nature of their interaction with their substrates, and the technical difficulty to reconstitute snoRNA release and rRNA folding events mediated by RNA helicases in vitro. Also, structural investigations are often challenging owing to flexible helicase domains and their association with flexible elements of pre-ribosomal particles. Considering their presumed key functions in rRNA folding, the functional characterization of RNA helicases in ribosome biogenesis will without doubt represent an important research line in the ribosome biogenesis field in future years.

## Supplementary Material

Supplemental Material
