# Supplementary figures and images for: RNA folding and functions of RNA helicases in ribosome biogenesis
Source: RNA Biol. 2022 Jun 9;19(1):781–810. doi: 10.1080/15476286.2022.2079890 (PMC9196750; doi:10.1080/15476286.2022.2079890)

# Supplementary Figure S1

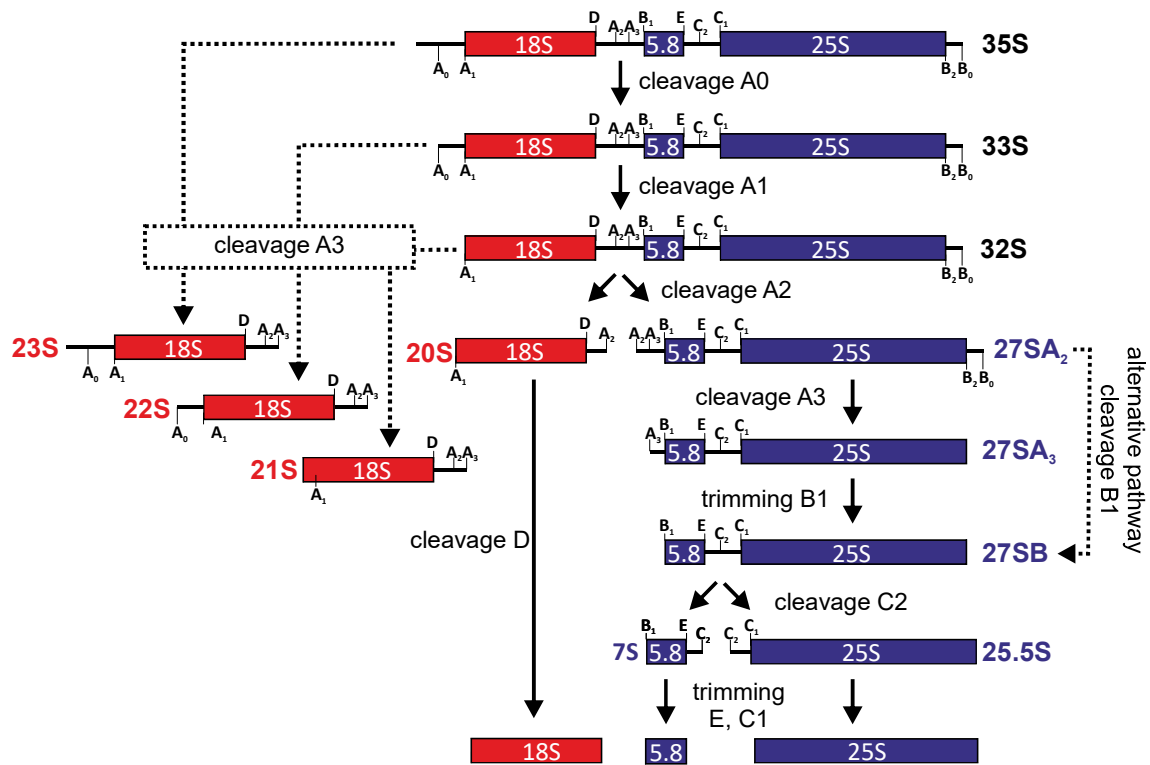

## Supplementary Figure S2

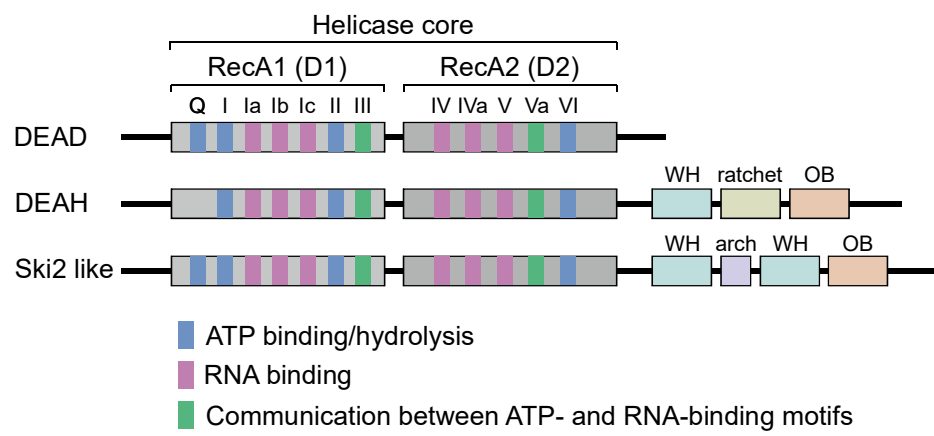

Supplement: Supplemental Material [file KRNB_A_2079890_SM4051.pdf]
